# Coordinated Residue Motions at the Enzyme–Substrate Interface Promote DNA Translocation in Polymerases

**DOI:** 10.1021/jacs.5c05888

**Published:** 2025-06-17

**Authors:** Alessia Visigalli, Enrico Trizio, Luigi Bonati, Pietro Vidossich, Michele Parrinello, Marco De Vivo

**Affiliations:** † Laboratory of Molecular Modeling & Drug Discovery, 121451 Istituto Italiano di Tecnologia, Via Enrico Melen 83, 16142 Genoa, Italy; ‡ Atomistic Simulations, Istituto Italiano di Tecnologia, Via Enrico Melen 83, 16142 Genoa, Italy

## Abstract

The translocation of DNA in polymerase (Pol) enzymes is a critical step for Pol-mediated nucleic acid polymerization, essential for storing and transmitting genetic information in all living organisms. During this process, the newly elongated double-stranded DNA has to shift along the Pol enzyme to recreate the initial configuration at the metal-aided reactive center, where nucleotide addition can occur recurrently at every catalytic cycle. Double-stranded DNA translocation, therefore, allows the enzyme to add one more nucleotide to the growing strand, complementary to the template strand, without the enzyme dissociating from the DNA. Yet, the dynamic mechanism by which the Pol·DNA complex accomplishes DNA translocation remains poorly understood at the atomistic level. Here, leveraging recent structural data on DNA polymerase η (Polη), we elucidate its translocation mechanism, which we show to occur via an enzyme motion where the shift of Polη is asynchronous along the two DNA strands. Through equilibrium molecular dynamics and deep-learning-guided enhanced sampling simulations, we found that such a mechanism relies precisely on a set of positively charged residues of the enzyme that operate in a coordinated way at the Polη·DNA interface. Moving like screen wipers, such a dynamic mechanism of these residues promotes DNA translocation. These findings now offer new avenues to comprehend further such a complex yet fundamental dynamic process for DNA polymerization.

## Graphical abstract

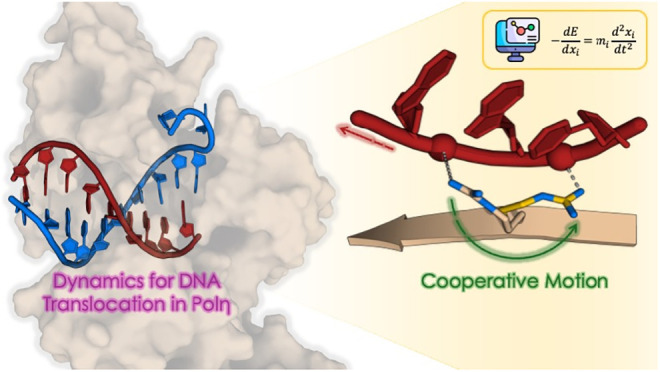

## Introduction

The translocation of DNA in polymerases (Pols) is an essential molecular process in cells, enabling DNA replication and transcription.^1,2^ This process occurs at every catalytic cycle in Pols after adding an incoming nucleotide (dNTP) to the 3′ primer terminus of the growing DNA strand (Figure 1A).^3–5^ That is, the DNA substrate translocates by one base pair at every catalytic cycle in Pols, resetting the active site so that it is free to add a newly incoming dNTP at the next Pol catalytic cycle.^6,7^ This molecular motion thus consists of a sliding motion of the newly elongated double-stranded DNA and the Pol enzyme.^8–10^ However, such a fundamental and complex dynamic mechanism for DNA translocation within the Pol enzyme remains largely unclear.

**Figure 1:**
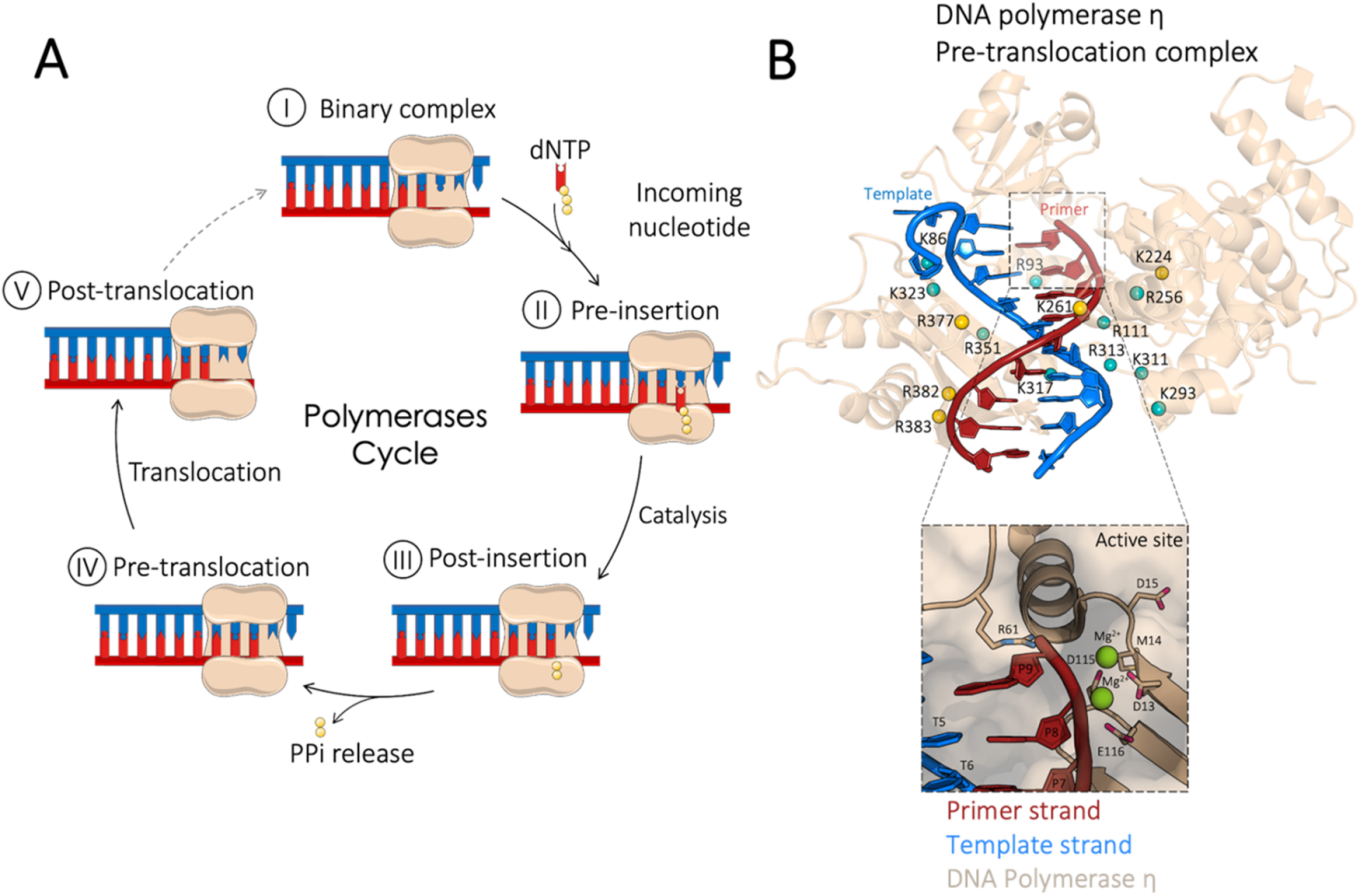
(A) Schematic representation of the Polη catalytic cycle. (I) the binary complex Polη·DNA_n_, (II) the ternary complex with the nucleotide located in the active site, (III) the binary complex formed after catalysis, (IV) the binary complex with the active site occupied and without the ions and the PPi, (V) the binary complex after the translocation. (B) Crystal structure of the DNA polymerase η (Polη) in the pre-translocation state (PDB ID: 4ECX). The key positive residues interacting with the DNA are shown in spheres and colored according to the strand they interact with (template: teal; primer: gold). Protein and nucleic acid are represented in cartoon, with different colors for the primer strand (red) and the template strand (blue). Close view of the active site, where the primer strand (red) is in cartoon representation, the active site residues are in sticks, and the magnesium ions are in spheres.

Here, we investigated DNA translocation in Polη, a low-processive enzyme belonging to the Y family DNA Pols,^6,11^ which is a promising drug discovery target for cancer.^12–16^ Polη represents an ideal model for understanding DNA translocation as it is highly characterized experimentally,^17–19^ with multiple ternary Pol·DNA·dNTP complexes showing the protein embracing the DNA substrate with all four subdomains: palm, fingers, thumb, and little finger (Supplementary Figure S1).^14,18^ Notably, all these four subdomains are prealigned for dNTP binding and catalysis.^20–22^ The palm subdomain contains the catalytic triad (DED) that coordinates the two metal ions (Mg^2+^) at the catalytic center. The finger subdomain lays upon the incoming nucleotide, with the highly flexible and conserved Arg61 stabilizing the catalytic transition state and promoting the exit of the leaving group pyrophosphate (PPi) after nucleotide addition.^23–25^ The thumb and the little finger subdomains surround the double-strand DNA, anchoring it through positively charged residues at the Pol·DNA interface.^14,26,27^ Importantly, recent time-resolved X-ray structures of human Polη have revealed the ternary Pol·DNA·dNTP complex in the course of DNA synthesis, thereby also enlightening an atomic picture of the starting and final configurations during DNA translocation.^18^ However, these static structures show a minimal overall structural rearrangement of Polη, passing from the starting to the final configuration during DNA translocation, making it hard to understand how DNA translocation is achieved mechanistically.

In this regard, two mechanistic models have been proposed to describe Pols translocation.^28,29^ The first is the “power-stroke” model, where the PPi release promotes significant structural changes in the enzyme that help translate the DNA substrate away from the active site. Such conformational changes are forceful and deterministic, triggering the Pol enzyme shift along the DNA strand. For instance, in A-family Pols such as Pol I, the close-to-open O-helix motion and the Tyr714 conformational change are proposed as the translocation trigger.^30^ Alternatively, the “Brownian-ratchet” model has been proposed to explain the translocation mechanism in Pols, based on the fast equilibrium between pre- and post-translocation states. In this model, thermal fluctuations, inducing mechanical force and motion, allow the enzyme to shift between initial and final translocation states randomly. These nonequilibrium fluctuations continue until nucleotide binding locks the system into the post-translocation state, acting as a ″ratchet″ stabilizing the system in a given state. Also here, the significant conformational motion of a specific helix in the finger subdomain, involving precise residues at the DNA·helix interface, represents the main dynamical trigger to promote DNA translocation. For instance, the Phi29 DNA Pol translocation mechanism is compatible with the Brownian-ratchet model, according to recent experimental evidences.^31,32^

However, these two mechanistic models apply only to Pols that show significant conformational rearrangements of the overall structure upon translocation. Polη, instead, lacks such major structural rearrangements. Indeed, the root-mean-square-deviation (RMSD) of the catalytic core differs by only 0.15 Å comparing the X-ray structures of the pre- and post-translocation states (PDB ID: 4ECX and 4ED8, respectively; Supplementary Figure S2). At the same time, no mobile structural elements show significant conformation shifts comparing pre- and post-translocation states. Intriguingly, this evidence suggested that Polη may operate through a different yet unknown translocation mechanism.

By integrating high-resolution X-ray crystallographic structures with microsecond-long molecular dynamics (MD) simulations and deep-learning-guided enhanced sampling techniques, we provide a comprehensive, atomistic characterization of the DNA translocation mechanism in the wild-type system of Polη. Even though crystallographic data offer invaluable static snapshots of Polη·DNA complexes captured in specific conformational states, they inherently lack information about the dynamic processes that govern enzymatic function. To bridge this gap, we employed MD simulations initiated from experimentally resolved structures, allowing us to explore the conformational landscape connecting the pre- and post-translocation states. This integrative approach reveals a previously uncharacterized, stepwise, and asynchronous translocation mechanism, in which DNA advances through the polymerase active site via a coordinated sequence of conformational rearrangements. These motions are driven by a set of strategically positioned, positively charged residues at the Polη·DNA interface, whose synchronized motion facilitates the directional movement of the DNA strand. Notably, the residues involved are functionally relevant and evolutionarily conserved across Y-family polymerases, suggesting that this mechanism may represent a general translocational strategy within the family.

## Results

### Molecular Dynamics Simulations Unravel the Coordinated Motion of Positive Residues at the Polη·DNA Interface

Here, we performed classical molecular dynamics simulations (MD) to investigate the wild-type Polη·DNA complex in both pre-translocation (PRE) and post-translocation states (POST). Our simulations revealed that, despite the overall structure of the complex remaining stable, key positively charged residues at the Polη·DNA interface exhibit dynamical rearrangements, influencing their interactions with the DNA substrate. These fluctuations, observed in both the protein-primer (Polη·Pi) and protein-template (Polη·Ti) interfaces, suggest a role in facilitating DNA translocation in Polη. By comparing the PRE and POST states, we found that some positively charged residues in the PRE state can flip, adopting a conformation that matches those in the POST state, indicating a spontaneous tendency toward translocation.

#### Pre-translocation State

First, we ran multiple replicas of classical molecular dynamics (MD) simulations of the wild-type Polη·DNA complex in the pre-translocation state (PDB ID: 4ECX; ∼2 μs, three replicas). This state corresponds to the post-catalytic complex after incorporating the incoming nucleotide. The Polη·DNA binary complex is stable in the pre-translocation state (PRE) for the entire simulation, with an average RMSD value for the heavy atoms of 2.5 ± 0.7 Å (Supplementary Figure S3). The movement of the DNA substrate is well described by the RMSD of the phosphates of the double helix, which is 2.3 ± 0.7 Å for the template strand and 2.4 ± 0.9 Å for the primer strand (Supplementary Figure S4). Despite the overall stability, the Polη·DNA system underwent conformational fluctuations coordinated with the DNA motion, which affected a few residues and their interactions with the surroundings. We noticed that at both the Polη·Pi and Polη·Ti interfaces, a few positively charged residues, i.e., arginines and lysines, oscillated significantly, changing their initial orientation and conformation, often breaking their starting interactions with the double-strand DNA (dsDNA). In particular, we identified 17 positively charged residues at the Pol·DNA interface that showed significant conformational fluctuations, 6 at Polη·Pi and 11 at Polη·Ti, which we discuss in the following.

In the PRE state, we could identify 6 residues (Arg61, Lys224, Lys261, Arg377, Arg382, and Arg383) interacting with the primer strand (Figure 2A, left panel). Remarkably, our simulations revealed that Arg382 and Arg383 underwent significant side chain rearrangements, breaking their initial interactions with the primer strand. The side chain alternated its contacts with two different phosphates of the primer strand (see Figure 2B). Arg382′s fluctuations led to a distance increase to P2 (from 4.9 ± 0.9 Å to 10.1 ± 0.5 Å) while also forming hydrogen bonds with the base T9 (4.5 ± 0.6 Å) or with P3 (2.9 ± 0.2 Å). Arg383 interacted with either P2 (5.2 ± 0.7 Å) or P3 (4.9 ± 0.4 Å), thanks again to its side chain flipping. Notably, Arg382 and Arg383 were not bound to the primer strand in the crystal structure. The interactions of Lys224 and Arg377 and the primer showed a different interaction network and conformational dynamics (Supplementary Figure S5). Here, these positive residues, which initially interacted with the DNA substrate, could flip to interact with a negatively charged residue of the protein, decreasing the number of electrostatic contacts with the DNA strand (Figure 2C, left panel). In particular, Lys224 interacted with P8 (4.0 ± 0.4 Å) or, after flipping, with the active site residue Glu116 (3.5 ± 0.3 Å, Supplementary Figure S5). Arg377 also fluctuated, approaching P4 (5 ± 1 Å) and transiently interacting with Asp375 (4.1 ± 0.2 Å, Supplementary Figure S5). On the other hand, Lys261 remained bound to the phosphate P5 for most simulations (4 ± 1 Å; PDB ID: 4ECX, 4.02 Å, Supplementary Figure S5). Finally, Arg61 could bind the phosphate P9, assuming the so-called A-conf (present in the 15% of our simulation and the crystal structure; PDB ID: 4ECX, 4.9 Å) or interact with T3, assuming the so-called C-conf (present in the 72% of our simulation).^23^ Notably, our previous simulations on the catalytic role of this arginine identified the importance of its spatial orientation in the native C-conf to promote the release of the pyrophosphate adduct (PPi+Mg_B_+Mg_C_) after the catalysis.^23^

**Figure 2:**
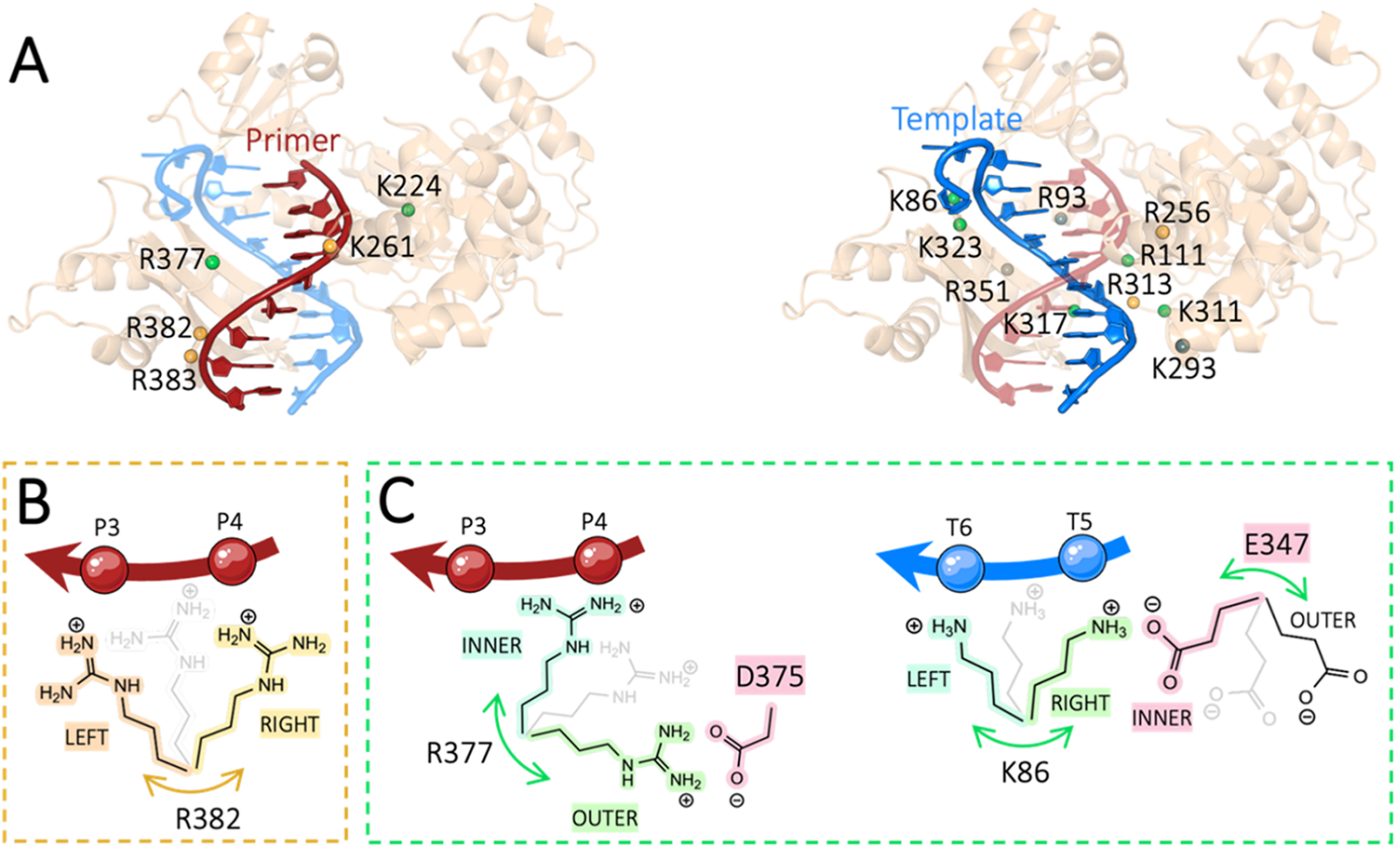
(A) Overview of positive residues at the Polη·DNA interface. On the left, the positive residues interacting with the primer strand (red). On the right, the positive residues interacting with the template strand (blue). The positively charged residues are color-coded according to the oscillatory motion explained in panels (B) and (C). (B) Schematic representation of the motion of the R382 side chain, which transiently interacts with two different phosphate atoms of the primer strand. (C) Schematic representation of R377, which changes its initial inner conformation to an outer one, unbinding the primer phosphate P4 in favor of the negative residue D375 (left panel). Another oscillatory motion is schematically represented for K86, which alternatively interacts with the phosphates T6 orT5 and E347 (right panel).

We identified 11 positively charged residues that anchored the template strand to the enzyme via electrostatic interactions (Figure 2A, right panel). It is worth noting that these are nearly twice as many as those anchoring the primer strand. Here, we found similar fluctuations as those of the residues interacting with the primer strand. For instance, Arg313 interacted with the base T8 during the simulation (5.29 ± 0.9 Å), despite maintaining stable interactions also with T9 (4.6 ± 0.8 Å, Supplementary Figure S5). Then, we found five residues (Lys86, Arg111, Lys311, Lys317, and Lys323) that interacted transiently with the template strand and/or with a protein residue (Figure 2C). In particular, Lys86 and Arg111 could contact the template strand thanks to the side chain flipping of a close negative protein residue (Figure 2C, right panel). Lys86 interacted with Glu347 (3.4 ± 0.2 Å) and T6 (4.1 ± 0.2 Å). However, Lys86 also transiently interacted with the phosphate T5 (from 6.6 ± 0.3 to 3.9 ± 0.3 Å). This conformational change happened after the flipping of the Glu347 side chain (from ∼100° (inner) to ∼300° (outer); see Supplementary Supplementary Figure S6 and Movie 1 in Supporting Information). Lys293 fluctuated near T10 (3.9 ± 0.3 Å) and T11 (3.8 ± 0.3 Å, Supplementary Figure S5). In the same way, Arg111 could transitorily interact with the phosphate T7 through a water molecule (6.6 ± 0.4 Å) or with Ser96 (distance measured with OH = 3.6 ± 0.2 Å). Arg111 flips its side chain frequently (from ∼ 110° (DNA bonded) to ∼ 20° or ∼ 350° (protein bonded), see Supplementary Figure S7), at times interacting with the phosphate T8, again with the help of a bridge water molecule (6.4 ± 0.5 Å). On the other hand, in our simulations, Lys311, Lys317, and Lys323 could break their initial interaction with the template phosphate, reaching a protein residue (Figure 2C, left panel). These residues fluctuated significantly as they sat in a solvent-exposed area of the protein (Figure 2A, right panel). Lys311 interacted with T9 (3.9 ± 0.3 Å) or with Asp308 (3.6 ± 0.3 Å). Lys317 underwent a conformational switch from T9 (4.0 ± 0.3, PDB ID: 4ECX, 4.02 Å) to Ser432 (3.5 ± 0.8 Å). Lys323 interacted with Tyr39 (from 4.2 ± 0.6 Å to 5.1 ± 0.9 Å) and T5 (from 3.6 ± 0.2 Å to 5.7 ± 0.6 Å) during the dynamics and approached the phosphate T4 (from 5.7 ± 0.7 Å to 4.7 ± 0.9 Å). Then, Arg371 and Lys376 transiently interacted with the template junction. During the simulations, they both got closer to T2 (from 13 ± 2 to 8 ± 3 Å and from 16 ± 4 to 12 ± 5 Å, respectively - Supplementary Figure S5). Simultaneously, the little finger subdomain exhibited a motion relative to the finger, widening the cavity to accommodate the template junction. Finally, we found two residues (Arg93 and Arg351) that maintained stable interactions with the template for the whole simulation (Supplementary Figure S5). Arg93 stably interacted with T6 (5.0 ± 0.3 Å; PDB ID: 4ECX 5.23 Å) and T7 (4.6 ± 0.4 Å, PDB ID: 4ECX 4.61 Å), while Arg351 maintained its interaction with T7 (4.2 ± 0.2 Å). Overall, we note that despite the residues anchoring the template strand being more numerous than those anchoring the primer (11 with template vs 6 with primer), in these 11, there are fewer protein residues able to flip their side chain to interact with a different phosphate atom of the template substrate.

#### Post-Translocation State

To analyze Polη·Pi and Polη·Ti at the end of the translocation process, we run additional equilibrium MD simulations of the wild-type Polη·DNA complex in the post-translocation state (POST, PDB ID: 4ED8; ∼ 2 μs for three replicas). The POST state is a Polη·DNA binary complex where the DNA substrate, if compared to the PRE state, is shifted by one base pair, thus leaving the active site empty and ready to bind the next incoming nucleotide (Supplementary Figure S8). Even if this Polη·DNA complex showed no significant differences in the overall backbone stability compared to the PRE state (paragraph above, see Supplementary Figure S9), the interface positive residues interacted with the DNA with a different interaction pattern from that observed in the starting configuration of the PRE state (Figure 3A). Interestingly, simulating the POST state, we noticed similarities and differences with respect to the PRE state. We first compared the conformations of positively charged residues in the post-translocation state with those observed in the pre-translocation state, focusing on residues that exhibited dynamic rearrangements. This analogy was aimed at identifying early structural signatures of the transition. In the post-translocation state, Arg382 and Arg383 displayed dynamic conformational flipping, interacting with different phosphate atoms of the primer strand (Figure 2B). Specifically, Arg382 remained in close contact with P3 (4.4 ± 0.2 Å), while Arg383 interacted alternatively with P3 (5.9 ± 0.6 Å) and P4 (4.9 ± 0.3 Å), similar to what was observed in the pre-translocation state. This suggests that their movement during PRE was already prone to evolve to the interaction network of the POST state. Also, our simulations revealed that Lys261 maintained its interaction with phosphate P5 in the post-translocation state, consistent with its behavior in the pre-translocation state. This indicates that this residue is relevant in stabilizing the DNA after translocation (Figure 3B). Our simulations also revealed similar results involving the positive residues interacting with the template strand (Figure 3), which mostly resembled the conformations freely adopted in the pre-translocation simulations (Supplementary Figure S10). For instance, Lys86, Arg111, Lys293, Lys323, and Arg371 adopted similar interactions in both states (Figure 3B). Arg313, which in the pre-translocation state had a tendency to interact with both T8 and T9, here is interacting with T8 (4.4 ± 0.3 Å) (Figure 3B). Notably, some residues differ in interactions with respect to what we observed in the PRE state. For instance, Lys224 and Arg377 were involved in stable interactions with the DNA backbone in the pre-translocation state simulations, keeping their positions without undergoing significant rearrangements (Supplementary Figure S5). Despite this, we found that Lys224 and Arg377 interact differently with the DNA substrate in the POST state simulations (Supplementary Figure S10).

**Figure 3:**
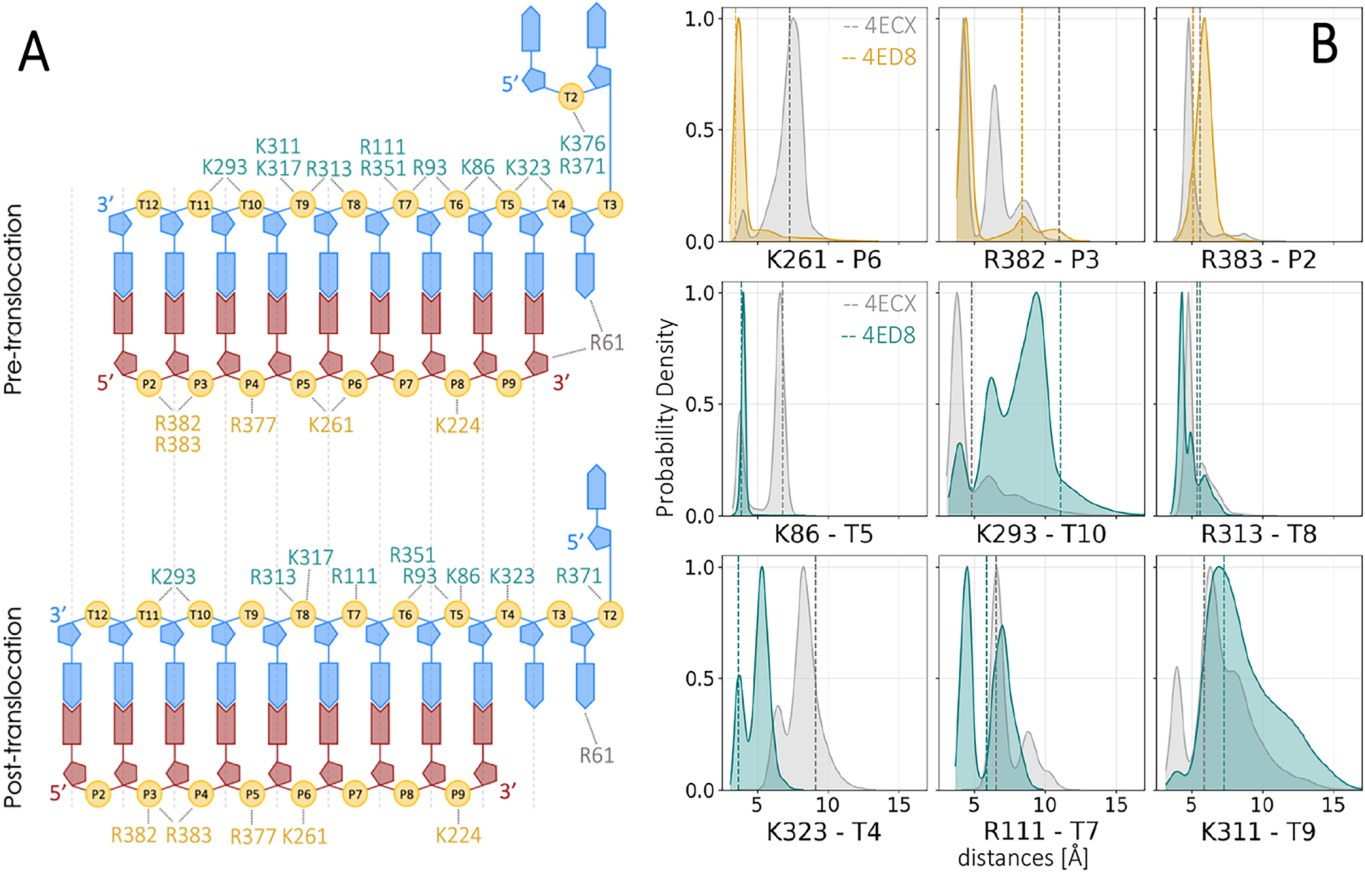
(A) Schematic representation of the Polη·DNA interactions in the pre-translocation (upper panel) and post-translocation (lower panel) state. The primer (red) and template (blue) strands are shown, with positive residues labeled in gold (primer-side contacts) and teal (template-side contacts). (B) Probability distributions of key residue–DNA distances, highlighting the conformational dynamics in the pre-translocation (gray curves) and post-translocation (colored curves) state. Distances for primer-side interactions (gold) and template-side interactions (teal) are shown, indicating the capability of some residues to reach the post-translocation conformation during the pre-translocation dynamics. The dashed lines represent the values of each distance in the X-ray structures (pre-translocation: PDB ID: 4ECX, gray; post-translocation: PDB ID: 4ED8, colored according to the strand interaction).

Therefore, our equilibrium simulations suggest some residues of the Polη·DNA system in the pre-translocation state are prone to evolve toward the post-translocation state after nucleotide addition. Indeed, the conformational changes of positively charged residues in the pre-translocation state match their final post-translocation conformations, supporting their role in facilitating DNA movement. However, the complete DNA translocation could not be observed within the conformational landscape explored, likely due to the limitation of the thermal fluctuations of equilibrium MD simulations.

### Enhanced Sampling Simulations Revealed that DNA Translocation Occurs via an Asynchronous Stepwise Mechanism

While classical MD simulations of the PRE and POST states provided valuable insights into general behaviors at the Polη·DNA interface, they were inherently limited in their ability to sample rare events, such as DNA translocation, which involve crossing significant free energy barriers. To promote such transition, we thus performed a set of enhanced sampling simulations using the On-the-fly Probability Enhanced Sampling (OPES) method combined with two machine-learning collective variables (MLCVs, see Methods section).

In the first stage, we aimed to explore the conformational landscape outside the metastable states. To this end, we exploited the information from the classical MD simulations to train a classifier-like MLCV (Deep-LDA CV, see Methods section). We used it to perform multiple OPES simulations starting from PRE and POST states (Supplementary Figure S11). In this way, we collected ∼ 20 μs of independent trajectories, observing 81 PRE ↔ POST successful conformational transitions, as confirmed by monitoring the DNA RMSD during the simulations (see Supplementary Figure S12 and Methods section)

Interestingly, within the Polη·DNA complex, we found that during the PRE ↔ POST conformational transitions, the two DNA strands translocate asynchronously, defining two mutually exclusive pathways (see Supplementary Figure S12). Each path is characterized by an intermediate, either INT1 or INT2 (fully described in Figure 4). The primer strand is fully translocated in the first intermediate state INT1, showing the interface interactions typical of the POST state (Figure 4A). In fact, all of the six positive residues interacting with the primer strand (Lys224, Lys261, Arg377, Arg382, and Arg383; see previous section) showed the same interactions with the primer strand that we found in POST during the equilibrium MD simulations. For instance, Lys224 stably interacts with P9 (3.9 ± 0.3 Å), and Arg383 is oscillating between P3 (4.4 ± 0.3 Å) and P4 (4.6 ± 0.3 Å). By contrast, the template strand maintained the same position and interactions that we found in PRE equilibrium MD simulations. Here, all 11 positive residues show the same interactions found in the PRE state. For instance, Lys86 interacts with T5 (3.8 ± 0.2 Å), and Arg313 is fluctuating between T8 (4.3 ± 0.3 Å) and T9 (4.6 ± 0.2 Å).

**Figure 4:**
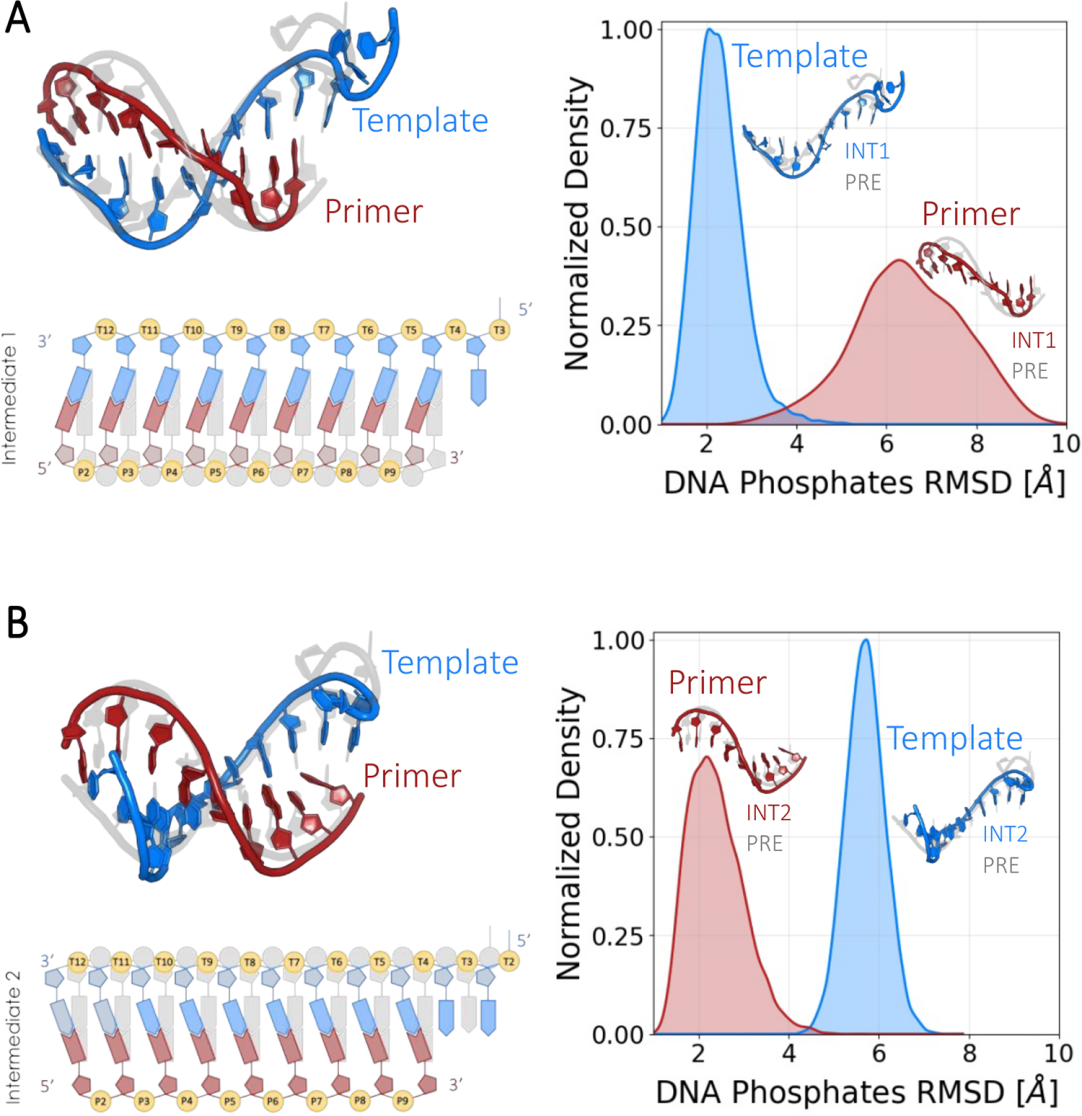
Description and classification of the two intermediate states. (A) Intermediate state 1 (INT1). On the left, the structural and the schematic representation of the nucleic acid in INT1. Here, the primer strand (red) is shifted by one base pair with respect to the pre-translocation state (gray). By contrast, the template strand (blue) is in the same position as in the pre-translocation state. On the right, the probability distributions of RMSD values for DNA phosphates in the primer (red) and template (blue) strands, comparing INT1 with the pre-translocation (PRE) state. (B) Intermediate state 2 (INT2). On the left, the structural and schematic representation of the nucleic acid in INT2. Here, the template strand (blue) is shifted by one base pair with respect to the pre-translocation state (gray). By contrast, the primer strand (red) is in the same position as in the pre-translocation state. On the right, the probability distributions of RMSD values for DNA phosphates in the primer (red) and template (blue) strands, comparing INT2 with the pre-translocation (PRE) state.

The other intermediate state, INT2, is specular with respect to INT1 (Figure 4B). Here, the primer strand maintained the interactions characteristic of the PRE state, with the positively charged residues at the Polη·Pi adopting the same configurations as in the PRE state MD simulations. For instance, Lys261 and Arg377 remained stably bound to P5 (3.9 ± 0.3 Å) and P4 (4.8 ± 0.3 Å), respectively, with distances comparable to those observed in the PRE equilibrium simulations (Supplementary Figure S5). At the same time, the template strand is translocated, assuming the interactions typical of the POST state. Indeed, the positively charged residues at the Polη·Ti adopted the POST state conformations. For example, Arg93, which in the PRE state interacted with T6 and T7, here shifted its interaction only to T6 (4.4 ± 0.3 Å), in agreement with what was observed in the MD simulations of the POST state (Supplementary Figure S10). Similarly, Arg313 is binding only to T8 (4.5 ± 0.4 Å), like in the POST state MD simulations. In summary, the two intermediates characterize two possible paths for translocation, showing a different shift order of the DNA substrate: either the primer translocates and then the template, as in INT1, or vice versa, as in INT2.

Having discovered these new intermediate states, we proceeded to the second stage, where we aimed to characterize the process in more detail. As a first step, we ran additional equilibrium MD simulations for INT1 and INT2 to test their stability (600 ns for INT1 and 1 μs for INT2; see Supplementary Figure S13). Then, we used this new information to design a more informative CV that could distinguish and promote both reaction pathways. To this end, we built a 2-dimensional CV following a multitask framework to exploit not only the information from the unbiased simulations but also from the reactive trajectory collected previously (see Supplementary Figure S14 and Methods section). With this approach, we obtained a two-dimensional CV space (CV1 and CV2) in which the four states were well-distinguished (see Figure 5), and we used them to perform new OPES biased simulations, collecting 20 transitions in 1.2 μs over the two paths.

**Figure 5:**
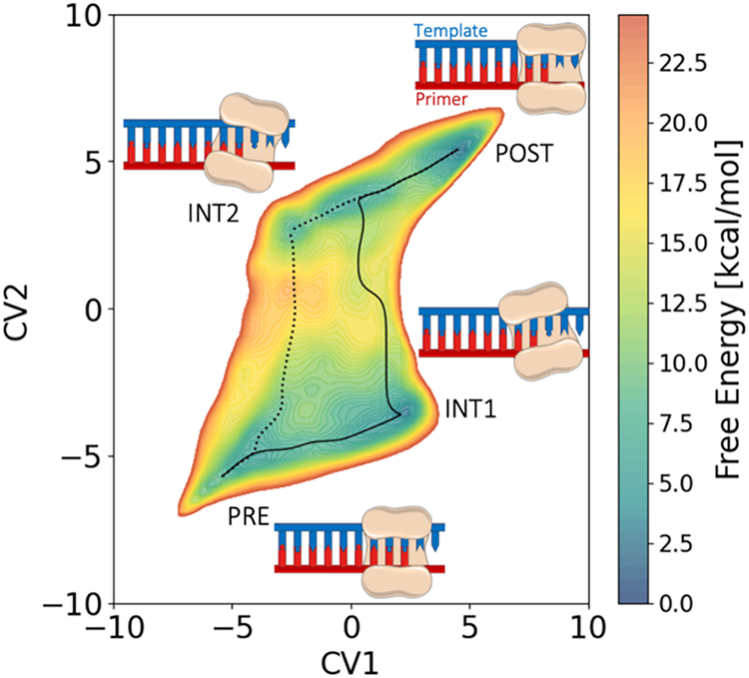
Free energy surface (FES) describing the two translocation paths: this FES represents two specific translocation pathways where Polη translocates from the PRE state to the POST state, passing through one of the two intermediate states (INT1 or INT2). The PRE state is located at the lower left corner of the FES, which is the starting point of the translocation process, with the DNA in a pre-translocated conformation. The POST state is positioned at the upper right of the FES, representing the final state of translocation, where the DNA has fully shifted to a post-translocation position. The dashed line traces the minimal energy path (pathway 1) between these states, showing how the system passes through energy barriers as it moves from the PRE state, through INT1, to the POST state. The dotted line highlights a different translocation pathway (pathway 2) where the polymerase transitions from PRE to POST, passing through INT2.

The first path involved the transition between PRE and POST via INT1 (Supplementary Figure S15A). Here, the PRE → INT1 transition was characterized by the changes in Polη·Ps interactions. For instance, the Lys224·P7 interaction was lost in favor of the formation of the Lys224·P8 interaction. The same applies to the other four positive residues (Lys261, Arg377, Arg382, and Arg383). Notably, the same conformational changes for all the other positive residues at the Polη·Pi were reversed in the opposite transition, i.e., INT1 → PRE. For example, the interaction Lys224·P8 was broken in favor of the formation of Lys224·P7. Finally, from INT1, the system evolved into POST, moving the template strand to its post-translocation position.

The second path, starting from PRE, passed through INT2 (Supplementary Figure S15B), thus translocating the template strand first. Then, the INT2 → POST transition occurred involving the primer strand translocation. As observed in the first path (PRE → INT1 transition, Supplementary Figure S15A), the interatomic contacts at the Polη·Pi got broken (Lys224, Lys261, Arg377, Arg382, and Arg383), allowing the growing strand movement and translocation.

Interestingly, these new simulations also highlighted the propensity of the primer strand to move first, compared to the template. Indeed, all the transitions starting from PRE evolved into INT1, and all the transitions from POST evolved into INT2. This qualitative dynamic behavior suggests that the cost needed to move the primer strand is lower than that required to move the template. To corroborate this observation further, we calculated the free energy surface (FES) involved in the translocation process (see Methods section).

On the FES of the wild-type system, the initial PRE state is a relative minimum, separated from INT1 by a barrier of 7.5 ± 0.5 kcal mol^–1^ (Figure 5). Then, in the INT1 → POST transition, the template strand must be translocated by one base pair, overcoming a barrier of 15.0 ± 0.5 kcal mol^–1^. these barriers explains why our equilibrium MD simulations could not sample this energetically costly transitions. Furthermore, the number of interatomic interactions that must be broken to move the template strand is almost double that of the primer strand. This path corresponds to the minimum energy path for Polη translocation, with an overall barrier of 15.0 ± 0.5 kcal mol^–1^ for the PRE → POST transition (Figure 5, solid line).

**Figure 6:**
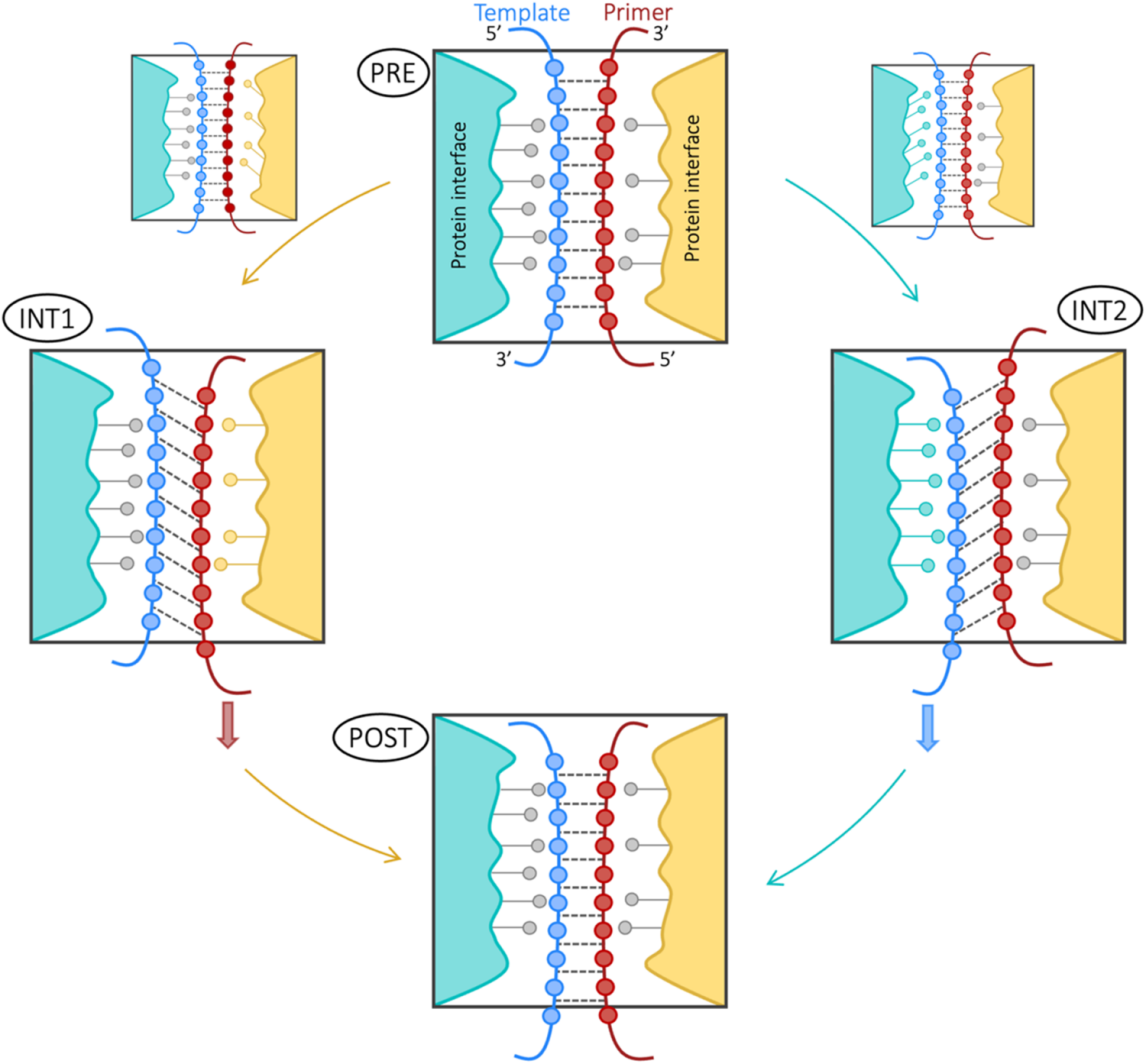
Schematic representation of the asynchronous DNA translocation mechanism in Polη.The transition from the pre-translocation (PRE) to the post-translocation (POST) state occurs via two mutually exclusive pathways, i.e., path1 on the left and path2 on the right. Each path is characterized by an intermediate state (INT1 or INT2). In INT1, the primer strand (red) is fully translocated, adopting the interactions typical of the POST state, while the template strand (blue) retains the PRE state configuration. Conversely, in INT2, the template strand is fully translocated, while the primer strand maintains its PRE state interactions. The final step leads to the fully translocated POST state, where both strands adopt their final positions. Arrows indicate the direction of strand movement.

Finally, we evaluated the FES for the alternative pathway, the PRE → INT2 transition (Figure 5, dotted line). This transition corresponds to the template strand translocation through a barrier of 17.0 ± 0.5 kcal mol^–1^. Once in INT2, the system must overcome a second energy barrier of 10.0 ± 0.5 kcal mol^–1^ to reach the POST state. Then, the primer strand must translocate to complete the transition, reaching its POST conformation. Again, our simulations revealed that moving the primer strand is energetically easier than moving the template. These results comply with the different number of positive residues anchoring each strand. That is, to evolve through this path, the system must overcome an overall barrier of 17.0 ± 0.5 kcal/mol^–1^ for the PRE → POST transition. This second path seems, therefore, unfavored compared to the one passing via INT1.

## Discussion

Recently, time-resolved X-ray structures describing the complete DNA synthesis were captured for the first time, characterizing the structure of human DNA polymerase η (Polη) enzyme before and after DNA translocation.^18^ Intriguingly, no significant conformational changes of the enzyme were observed between the initial and final states, with the corresponding crystal structures being mostly identical, apart from the shifted DNA substrate. Therefore, the dynamic mechanism governing DNA translocation in Pol enzymes remained unclear. Here, we report microsecond-long molecular dynamics (MD) and deep-learning-guided enhanced sampling simulations of the wild-type system of Polη, considering the initial and final state for DNA translocation.

During our multiple equilibrium MD simulations (∼2 μs for each replica), we first observed significant motions and structural rearrangements of some positive residues at the Polη·DNA interface. This dynamic motion suggested that those specific residues may help initiate DNA translocation, grasping the DNA double-strand and sliding it inside the enzyme. Therefore, we grouped such residues according to their particular interaction with either the primer or the template DNA strand. Remarkably, four of the six positive residues that anchor the primer strand (Arg61, Lys261, Arg382, and Arg383) underwent conformational changes in the pre-translocation state MD simulations. Indeed, these residues fluctuated in the initial state to transiently assume the conformations adopted in the post-translocation state (PDB ID 4ED8).

On the other hand, the template strand is anchored to the enzyme by eleven positive residues. Here, six over eleven (Lys86, Arg111, Lys293, Arg313, Lys323, and Arg371) fluctuated in the pre-translocational state simulations and transiently adopted configurations typical of the post-translocation state. These motions suggest the propensity of these residues to change their initial conformation in the pre-translocation state and, in doing so, likely assist DNA translocation in reaching the post-translocation state. Intriguingly, the coordinated motion of the side chain of these specific positively charged residues resembled the motion of screen wipers, which would assist the sliding of the newly elongated double-stranded DNA along the Pol from the initial to the final post-translocation state.

Then, we further investigated this mechanistic observation of a coordinated motion of such residues to assist DNA translocation using enhanced sampling MD simulations.^33^ Specifically, we sampled the translocation between the pre- and post-translocation state to energetically characterize DNA translocation in Polη and further analyze how the Polη·DNA interface residues would behave dynamically. Notably, we leveraged recent advances in machine-learning-based collective variables, which enabled the collection of 81 transitions connecting pre- and post-translocation states.^34^

We found two distinct pathways for DNA translocation, evolving the system from the pre- to post-translocation state (Figure 6). Both pathways showed an asymmetric stepwise mechanism with the formation of an intermediate, either INT1 or INT2, each along one of the two possible pathways. Remarkably, these intermediates drastically differed in their strand conformation in relation to DNA translocation. That is, the two strands adopted opposite conformations in INT1 and INT2. In INT1, the template strand was still in the pre-translocation state, while the primer strand had already shifted to the post-translocation state. Vice versa, in INT2, the primer strand was still in the pre-translocation state while the template strand was already shifted to the post-translocation state. Both pathways thus imply an asynchronous mechanism for DNA translocation. We then refined our initial CVs, also considering such intermediates (see Methods section), and generated a two-dimensional CV space where each intermediate state defines a possible translocation path. Notably, these two paths are mutually exclusive as they depend on the exact shifting order of the DNA strands.

The free energy surface (FES) computed upon this two-dimensional space confirmed the presence of two competitive paths (Figure 5). As a result, we found that the energy needed to move the primer strand first (INT1, path1, ∼7.5 kcal mol^–1^) is lower than the one required to translocate the template strand first (INT2, path2, ∼17.0 kcal mol^–1^). This disparity can be explained by looking at the number of positive residues anchoring each strand. Our results suggest that less energy is needed to move the growing strand because fewer interface bonds are present. By contrast, more energy is required to break the interactions between the protein and the template, which is somehow logical as this strand serves as a reference guide for polymerization. Our simulations do not explicitly model PPi release, so they cannot directly determine whether the translocation energy is chemically or thermally driven. Indeed, previous computational studies supported the separation between chemical and physical steps during Pols catalysis, suggesting that the two catalytic Mg^2+^ ions are released before the translocation occurs.^30^ It is also proposed that the presence of a third metal ion lowers the reaction barrier and promotes leaving group departure during catalysis.^35–39^ In our simulations, we noticed a few (two or three) ions that transiently bind in the vicinity of the catalytic site.^5^ However, it remains unclear if such transient binding events can somehow affect translocation. For instance, recent computational studies have pointed out the correlation between ATP binding and hydrolysis to translocation motions in helicases or motor proteins, suggesting the coupling between chemical and physical steps.^40,41^ Nevertheless, Polη does not undergo major conformational changes throughout its catalytic cycle,^18^ supporting that structural changes resulting from nucleotide binding are unlikely to propagate far from the active site

Notably, recent structural studies of other Pols captured the enzyme in an intermediate state analogous to INT1.^42^ In addition, in viral RdRPs, the template strand movement was proposed as the rate-limiting step, attributed to the positioning of the template junction.^43^ In our simulations, the RMSD of the primer strand increases during the PRE → INT1 transition (from 2.5 ± 0.6 to 6.1 ± 0.5 Å), in contrast to the template strand RMSD, which remains relatively stable (from 2.9 ± 0.5 to 3.3 ± 0.6 Å). Then, the INT1 → POST transition is characterized by better stability of the primer strand (from 6.1 ± 0.5 to 6.7 ± 0.4 Å) and the movement of the template strand (from 3.3 ± 0.6 to 6.2 ± 0.5 Å). The template junction is stabilized through stacking interactions at the interface between the finger and little-finger subdomains. In other words, the finger·little-finger interface helps accommodate the base of the template junction. In analogy, in Polη, two aromatic residues, Trp42 and Trp64, are located at this interface. These residues interact with the nucleic acid bases of the template junction, acting as a gating mechanism for substrate accommodation. Clearly, additional simulations involving mutated systems are needed to confirm this mechanistic hypothesis.^44^ It is, however, worth noting that all the Y-family Pols (including Polκ, Polι, Rev1, Dbh, and Dpo4) present at least one aromatic residue at the finger·little-finger interface (Supplementary Figure S16).^11,45–47^

Although the sequence of the catalytic core in Y-family Pols is well conserved, the little-finger subdomain shows high sequence variability (see Supplementary Table 1).^47^ Remarkably, however, we found that the five residues that anchor and stabilize the primer strand in Polη are structurally conserved among the protein family (see Figure 7), except for Arg377. This discrepancy depends on the position of this residue. In fact, the loop where this residue is located can be very long and flexible, such as in Polη and Rev1, or small and far away from the DNA substrate, like in Dpo4. By contrast, the positive residues at the finger·little-finger interface are conserved in Dpo4, Polκ, and Polι. In Rev1 and Dbh, there is a huge gap between finger and little-finger subdomains, which likely explains why there are fewer residues at the interface.^14^ The linker region is well-conserved among the Y-family despite the differences in the number of residues that make up this region. The observation that the little-finger subdomain, characteristic of the Y-family Pols, exhibits the least sequence conservation is particularly intriguing.^48^ Despite this variability, the near-complete conservation of positively charged residues within this subdomain strongly supports the hypothesis that these residues are crucial for function, in analogy to several other nucleic-acid-processing enzymes.^49^ Furthermore, the little-finger subdomain is absent in Pols that employ alternative translocation mechanisms, suggesting it is specifically adapted to facilitate this particular mechanism. Notably, the departure of the little-finger subdomain from the finger subdomain appears crucial for template strand translocation, which would otherwise be hindered.

**Figure 7:**
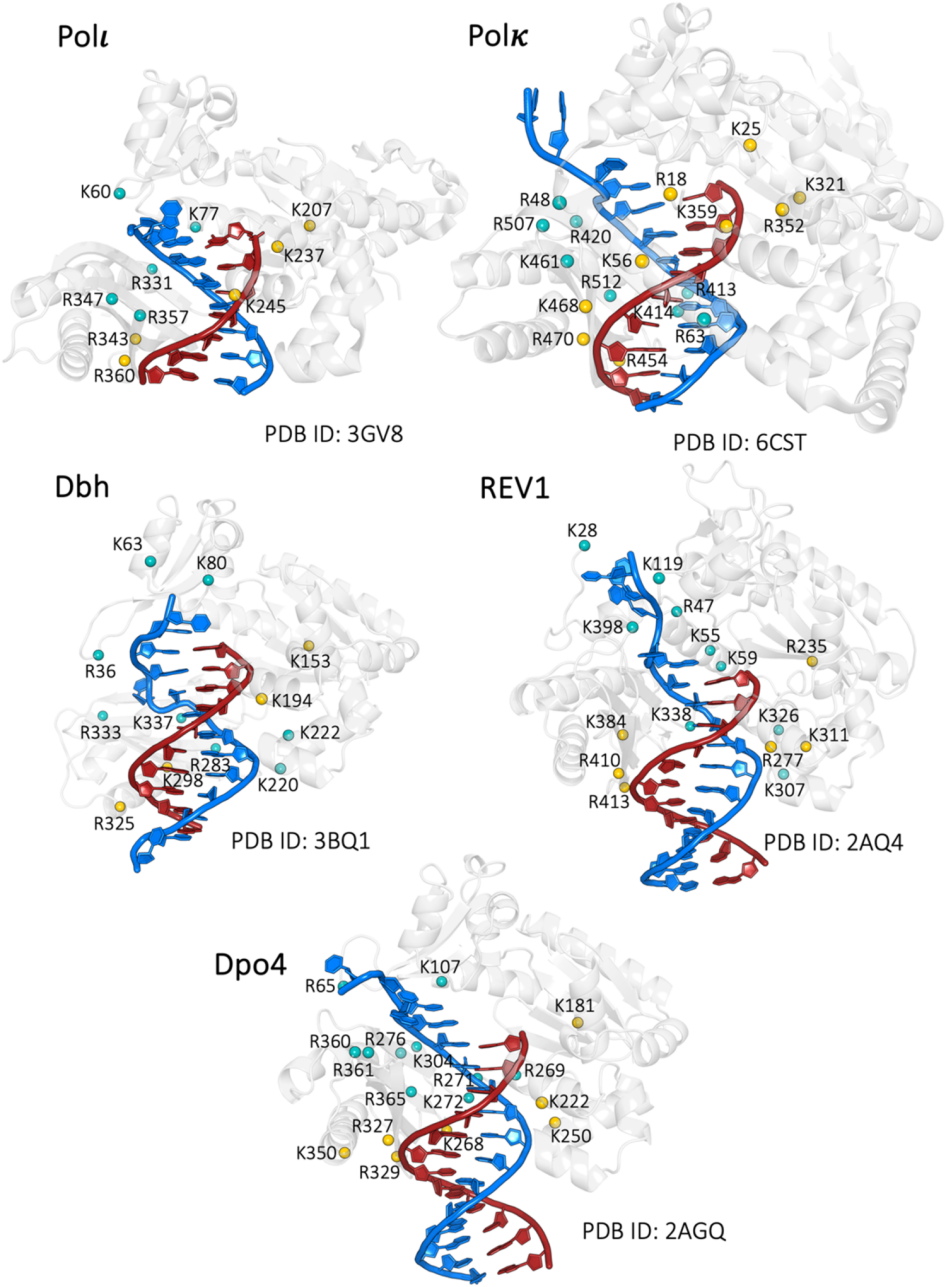
Structural comparison of positively charged residues interacting with DNA in Y-family polymerases. Crystal structures of Polι (PDB ID: 3GV8), Polκ (PDB ID: 6CST), Dbh (PDB ID: 3BQ1), Rev1 (PDB ID: 2AQ4), and Dpo4 (PDB ID: 2AGQ) highlighting positively charged residues (Arg and Lys) at the protein–DNA interface. DNA is shown in blue (template strand) and red (primer strand), while positively charged residues are represented as spheres (teal if they interact with the template strand; gold if they interact with the primer strand) and labeled. The structural alignment reveals conserved electrostatic interactions stabilizing the DNA substrate across different polymerases, despite the sequence variability in the little-finger subdomain.

Finally, we investigated whether other Pols may operate through such a stepwise translocation mechanism (Figure 7). For instance, Polι and Polκ show a comparable number of positive residues at the Pol·DNA interface (10 and 17, respectively). Also, none of the Y family members show a secondary structure element like the O-helix in the finger subdomain of A-family Pols. The closing motion of such O-helix in the finger subdomain at the end of the catalytic step is a possible trigger for DNA translocation in A-family Pols.^30^ Nevertheless, such O-helix (or an analogous structural element) is only conserved in some Pols, and this mechanism cannot be put into action in Polη. On the other hand, RNA polymerase RdRPs do not show minor conformational rearrangements typical of an open-to-close transition of a helix.^43^ In this system, recent crystal structures captured a translocation intermediate where the only growing strand was shifted, suggesting an asynchronous mechanism as the one observed in Polη.^42,50–52^

## Conclusions

Overall, our results elucidate a new translocation mechanism in DNA polymerase η (Polη), which is characterized by the coordinated motion of strategically located positively charged residues at the Pol·DNA interface. Such a coordinated motion allows them to grasp and slide the DNA substrate inside the Pol binding site. Based on our extensive simulations and free energy calculations, corroborated by a wealth of existing experimental data, we therefore proposed an asynchronous translocation mechanism involving two competitive paths for DNA translocation. The favored pathway is where the primer strand translocates first, followed by the template strand, which is anchored more strongly to the enzyme by several positive residues. Based on structural analyses and conservation, such a dynamic stepwise mechanism will likely be operated within the Y-family Pols. Ultimately, our findings propose a new mechanism for DNA translocation in Pols, offering experimentally testable predictions to help further clarify this fundamental and complex dynamic mechanism for DNA polymerization.

## Methods

### Overview

Here, we first summarize the elements of our methodology before discussing them in detail in the following paragraphs. In the preparatory stage, we set up the structures for our simulations, starting from the X-ray structures of PRE and POST translocation states, and we equilibrated them with respect to the final simulation conditions. In the production stage, we performed several μs of simulations.^53^ They included both classical molecular dynamics (MD) simulations and enhanced sampling ones based on the On-the-fly Probability Enhanced Sampling method (OPES) using two different sets of machine-learning collective variables (MLCVs) to drive our simulations.^54^ Initially, we trained a classifier-based CV (Deep-LDA CV) from information limited to the metastable basins to drive some first exploratory transitions. Then, we used the newly generated reactive data, which suggested the presence of two reaction pathways, to train a more informative two-dimensional CV using a multitask approach, which was used to perform the final production runs.

### Structural Models

We performed MD simulations on two different model systems: (i) wild-type pre-translocation binary state (PRE), based on the X-ray structure of the ternary complex (PDB ID: 4ECX; 1.74 Å); notably, the experimental structure includes both the reactants (Polη·DNA_n_+dATP) and product states (Polη·DNA_*n*+1_+PPi) of catalysis, with different occupancies: the reactants’ occupancy is 30%, while the products’ occupancy is 70%. The product state was used to build the model for the pre-translocation state, with the incoming nucleotide bonded to the growing filament. (ii) the wild-type post-translocation binary state (POST), based on the X-ray structure of the translocated binary complex (PDB ID: 4ED8; 1.52 Å); we replaced the TG mismatch with a TA base pair to pair the DNA sequence in the two models. Then, we removed Ca^+^ and Mg^2+^ ions and the PPi from the active site of both systems to favor translocation.^30,55^ Each system was solvated with a 12 Å layer of TIP3P water molecules. Counterions Na^+^ and Cl^–^ were added to neutralize the system and reach physiological ionic strength. The total number of atoms was ∼ 70000 for each system.

### Data and Software Availability

The used PDB files were downloaded from the RCSB Protein Data Bank (https://www.rcsb.org/). MODELER version 10.3 was used to model the two states, adding the missing residues to the protein (https://salilab.org/modeller/).^56^ tleap from AmberTools21 was used to create the topology of the system and to add the missing DNA bases.^57^ The GPU version of pmemd from AMBER20 was used to perform classical MD simulations (https://ambermd.org/).^58,59^ The training of the MLCVs has been performed using the open-source Python library mlcolvar (https://mlcolvar.readthedocs.io/en/stable/).^60^ The enhanced sampling simulations have been performed using the open-source plugin PLUMED 2.9 (https://www.plumed.org/doc-v2.9/user-doc/html/index.html), with the optional pytorch module of the code enabled and the support of the toolkit MDAnalysis.^61–63^ This, patched with the GPU version of pmemd of AMBER22,^59,64^ has been used to simulate the translocation in the wild-type system.^64^ All the structures in the figures have been produced using the educational version of PyMOL 3.1 (https://www.pymol.org).^65^

### System Equilibration

The MD simulations to equilibrate the system were performed using the GPU version of the pmemd module of AMBER 20.^66^ The AMBER-ff14SB force field was used for the enzyme,^67^ while the bsc1 force field was used for the DNA substrate.^68,69^ Monovalent ions were described using Li-Merz parameters,^70^ and the TIP3P model for waters.^71^ Long-range electrostatic interactions were calculated using the particle mesh Ewald method.^72^ Periodic boundary conditions in the three directions of Cartesian space were applied. A time integration step of 2 fs was used, and the lengths of all bonds involving hydrogen atoms were constrained using the SHAKE algorithm.^73^ To equilibrate the system, we first minimized the energy to relax the water molecules and the ions. Here, the X-ray-resolved atoms of the Polη·DNA complex were fixed with harmonic positional restraints with force constants of 1000 kcal mol^–1^ Å^2^ within AMBER. Then, the systems were heated from 0 to 310 K with an NVT simulation for ∼ 2 ns using the default AMBER Langevin thermostat,^74^ still constraining the enzyme and the DNA backbone with AMBER. Additionally, ∼ 3 ns of simulation in the NPT ensemble was performed with no restraints and a constant temperature of 310 K using the default AMBER Monte Carlo barostat and Andersen-like thermostat.^75,76^ Finally, we checked each wild-type system’s stability, performing three ∼ 2.0 μs-long independent replicas in the NPT ensemble. All the MD runs performed after the equilibration have also been performed in the NPT ensemble.

### Definition of Protein–DNA Contacts

Distances between positively charged amino acids and the DNA backbone were calculated to quantify protein–DNA interactions in pre-translocation and post-translocation states. For lysine residues, the Nζ atom of the side chain was used, while for arginine residues, the Cζ atom of the guanidinium group was considered. These distances were computed relative to the phosphate (P) atom of the DNA backbone. This approach was used consistently throughout the analyses, including those shown in Supplementary Figures S5 and S10.

### Deep-LDA CV

In the Deep Linear Discriminant Analysis (Deep-LDA) framework, the CV is expressed using a neural network that takes as inputs a set of physical descriptors and is optimized to maximally separate the metastable states (Supplementary Figure S17).^77^ The training requires a dataset with configurations from the metastable states with their corresponding labels, which can be easily obtained by running short unbiased simulations starting from each state. The parameters of the Deep-LDA CV are then optimized to maximize the Fisher’s ratio, which is the ratio of the variance between the states and the variance within them. To select the input descriptors, we first identified some ∼350 protein–DNA candidate contacts based on the PRE and POST states equilibrium MD simulations. We then performed a filtering, selecting only the contacts which (i) were present for at least 50% of one of the unbiased trajectories; (ii) that were significantly different between the PRE and POST states, eliminating those whose mean was within two standard deviations between states; and finally (iii) we retained one contact per each residue-base pair to reduce redundancy (Supplementary Figure S18). This resulted in a curated selection of 69 Pol·DNA contacts and, from each 2 μs-long equilibrium MD, 20000 data points per state were used to train the Deep-LDA model.

For the training, we used the Deep-LDA implementation available in the mlcolvar library.^60^ The neural network architecture used for the training was [69, 30, 20, 15, 10, 5] nodes/layer using the rectified linear unit (ReLU) activation function. The training was performed for 500 epochs with a learning rate of 0.0005, an L_2_ regularization of 10^–4^, a within-scatter regularization S_w_ of 0.05, and a Lorentzian regularization of 40. This resulted in a deep-learning CV (Deep-LDA CV) where the PRE and POST states were mapped at 1 and −1, respectively (Supplementary Figure S5).

### Multi-Task CV

The Deep-LDA CV was sufficient for enhanced sampling simulations to transit between the PRE and the POST states (Supplementary Figure S11). However, we observed that in these simulations, two different intermediates were sampled (INT1 and INT2). Thus, to properly describe these two reactive pathways for the translocation of the wild-type system, we increased the dimensionality of the CV space, designing a two-dimensional machine-learning CV, which we optimized in a semisupervised multitask framework (Multi-Task CV)^60^ to use the reactive trajectories previously generated to improve the overall quality of the learned CV space.^77^ This framework allows the optimization of a single set of CVs, leveraging information from datasets of different sources. To do so, using as inputs the same descriptors as for the Deep-LDA CV, we expressed our CVs as the latent space of an autoencoder neural network, which was optimized to achieve two different tasks: dimensionality reduction of the transition pathways (unsupervised learning) and classification of the different metastable states (supervised learning) (Supplementary Figure S14).^60,78^ The parameters of the autoencoder were optimized by minimizing the sum of two loss functions. The first one is the reconstruction loss ( $L_{\mathrm{rec}}$ ), which aims to learn a compressed representation that is able to reconstruct the original input. In our case, the unlabeled dataset used for this task was composed of ∼ 120000 configurations from the reactive trajectories generated using Deep-LDA.^77^

The second term in the loss function comes from the Deep Targeted Discriminant Analysis (Deep-TDA) loss acting on the latent CV space ( $L_{\mathrm{TDA}}$ ), which enforces that the data from the different metastable states are distributed according to a simple preassigned target distribution, in which the states are well-defined.^79^ In practice, this target distribution is defined as a mixture of Gaussians, one associated with each metastable state and characterized by their mean and standard deviation μ_tg_ and σ_tg_ positioned at different locations of the CV space. For this task, we used a dataset of ∼ 62500 configurations generated with classical MD for each of the four states explored from the system during the previous transitions (PRE, POST, INT1, and INT2) and labeled accordingly. By integrating the two losses, we obtained a more informative CV that provided a robust mapping of the system’s dynamics both inside and outside the metastable basins, effectively incorporating information from both labeled and unlabeled datasets (Supplementary Figure S14).

For the training, we used the Multi-Task CV framework available in the mlcolvar library.^60^ The architecture of the encoder network of the model was [69, 50, 30, 15, 5, 2] nodes/layer and symmetric for the decoder network [2, 5, 15, 30, 50, 69] nodes/layer and the shifted softplus activation was used for both networks. The training was performed for 500 epochs with a learning rate of 0.001. For the TDA part of the loss, we set the centers of the target Gaussians associated with the states to μ_tg_=([-6,-6], [-3,3], [3,-3], [6,6]) and their sigmas to σ_tg_=([0.2,0.2], [0.2,0.2], [0.2,0.2], [0.2,0.2]), for the PRE, INT2, INT1, POST states and the two components CV1 and CV2, respectively. This resulted in the two-dimensional MLCVs, composed by CV1 and CV2 in which the states PRE, INT1, INT2, POST, were mapped at [-6,-6], [3,-3], [-3,3], [6,6].

### Enhanced Sampling Method: OPES

To perform our enhanced sampling simulations, we used the On-the-fly Probability Enhanced Sampling (OPES) method, which can be seen as an improved version of the well-known Metadynamics algorithm.^80^ Their working principles are similar, both facilitating transitions across considerable free energy barriers by building an external bias potential aimed at filling the metastable basins to remove the kinetic bottlenecks that hinder sampling. However, in OPES, the construction of the bias is formulated in the more general framework of probability distributions, which allows for several improvements, e.g., faster simulations and convergence, and a few parameters to be set by the user. In practice, the OPES bias *V*_OPES_ is defined as a function of a set of few collective variables *s*, which are, in turn, function of the atomic coordinates and is optimized in a self-consistent way to drive the sampled distribution *P*(*s*) toward a target distribution *P*_tg_(*s*) in which sampling is easier

$$V_{\mathrm{OPES}}=\frac{1}{\beta }\;\log\left(\frac{p\left(s\right)}{p^{\mathrm{tg}}\left(s\right)}\right)$$

Here, we used as target the well-tempered distribution $P^{\mathrm{WT}}\left(s\right)\propto {\left[P\left(s\right)\right]}^{1/\gamma }$ that aims at broadening the *P*(*s*) according to the so-called biasfactor γ > 1 .

In our simulations, we employed the Explore variant of OPES implemented in the open-source plugin PLUMED, where, in practice, most internal parameters are automatically determined according to a few key parameters set by the user: the STRIDE of the bias update, the BARRIER which is expected to be overcome and, optionally, the initial SIGMA of the Gaussian kernels, which is then eventually adapted automatically with time.^54,81^

In this work, we first performed OPES on the one-dimensional Deep-LDA CV, collecting a large set (>80) of exploratory runs. At this stage, we set a barrier of BARRIER = 20 kcal/mol and a kernel deposition rate of STRIDE = 50000. In addition, to prevent the system from escaping the physical region of the CV (i.e., ∼ [−1, 1]), we set with PLUMED two repulsive walls at CV_LDA_ ≥ 1.5 and CV_LDA_ ≤ −1.5 with force constants of 40000 kcal mol^–1^ Å^2^.

We then performed a second set of OPES simulations using the two-dimensional Multi-Task CV (CV1, CV2), observing ∼ 20 in 1.2 μs over six independent replicas. Notably, our Multi-Task CV pushed the system through either of the two pathways, reaching the expected final state. For these simulations, we set the barrier to BARRIER = 20 kcal/mol, the deposition rate as STRIDE = 500, and the initial width of the kernels to SIGMA = 0.1. Also in this case, to prevent the system from escaping the physical region of the CV (i.e., ∼[−6, 6], [−6, 6]), we set with PLUMED two repulsive walls at [−7.5, 7.5], [−7.5, 7.5] with force constants of [40000,40000] kcal mol^–1^ Å^2^. In addition, to prevent breaking the hydrogen bonds between the DNA bases, we defined a set of repulsive walls at 4.5 Å with a force constant of 150 kcal mol^–1^ Å^2^ for each distance involved in those hydrogen bonds.

## Supplementary Material











## Data Availability

Trajectories are available upon request, while representative structures from all states (pre-translocation, post-translocation, intermediate state 1, intermediate state 2) and the input/output files used in PLUMED are available in AV’s GitHub repository (https://github.com/alevisigalli/PolETA_translocation).

## Notes

Further analysis of classical MD simulations; collective variables design; details on enhanced sampling free energy calculations (PDF)
Movies 1 and 2 captions (PDF)
Movie 1: Conformational Transitions of Human DNA Polymerase η During DNA Translocation. (MPG)
Movie 2: Asynchronous Translocation Pathway — Zoom on Arg382 and Arg383 Conformational Rearrangements. (MPG)
Input and output files used in the molecular dynamics and enhanced sampling simulations of DNA translocation by polymerase η (Pol η). These data support the analyses presented in our study, including the definition of a multi-task collective variable (CV) and the reconstruction of the free energy landscape. (ZIP)
