# Supplementary material for: Coordinated Residue Motions at the Enzyme–Substrate Interface Promote DNA Translocation in Polymerases
Source: J Am Chem Soc. 2025 Jun 17;147(26):22972–85. doi: 10.1021/jacs.5c05888 (PMC12232177; doi:10.1021/jacs.5c05888)
Supplement: Supplementary file 1 [file ja5c05888_si_001.pdf]

Coordinated residue motions at the enzyme-substrate interface promote DNA translocation in polymerases.

Alessia Visigalli[1], Enrico Trizio[2], Luigi Bonati[2], Pietro Vidossich[1],
Michele Parrinello*[,2], Marco De Vivo*[,1]

[1] Laboratory of Molecular Modeling & Drug Discovery, Istituto Italiano di Tecnologia,
Via Enrico Melen 83, 16142, Genoa, Italy

[2]Atomistic Simulations, Istituto Italiano di Tecnologia,
Via Enrico Melen 83, 16142, Genoa, Italy

Corresponding authors:
Marco De Vivo, marco.devivo@iit.it
Michele Parrinello, michele.parrinello@iit.it

Supporting Information

Table of Contents

Supplementary Figures

Figure S1: Structural representation of the Polη, highlighting its subdomain organization. 4

Figure S2: Structural superimposition of the catalytic core in pre-translocation (PDB ID 4ECX, wheat) and post-translocation (PDB ID 4ED8, purple) states X-ray structures. 5

Figure S3: Time evolution of the Root-Mean-Square Deviation (RMSD) of the heavy atoms in the pre-translocation state over a 2 μs simulation...6

Figure S4: Root-Mean-Square-Deviation (RMSD) of the template strand (blue) and the primer strand (red) in the pre-translocation state. 7

Figure S5: Probability distributions of key residue–DNA distances in the pre-translocation state...8

Figure S6: Dihedral angle $\chi1$ of Glu347 in the pre-translocation state classical MD simulation. ..10

Figure S7: Probability density distribution of the dihedral angle χ2 of Arg111 in the pre-translocation state. 11

Figure S8: Comparison between the pre- and post-translocation states active site. 12

Figure S9: Time evolution of the Root-Mean-Square Deviation (RMSD) of the heavy atoms in the post-translocation state over a 2 μs simulation. 13

Figure S10: Probability distributions of key residue–DNA distances in the post-translocation state. 14

Figure S11: Evolution of the On-the-fly Probability Enhanced Sampling (OPES) 1D collective variable (CV) over time.[4–6]..15

Figure S12: Root-Mean-Square-Deviation (RMSD) of the template strand (blue) and the primer strand (red) over time during the translocation..16

Figure S13: Classical MD simulations of the two intermediate states. 17

Figure S14: Multi-Task framework...18

Figure S15: Two transition pathways in the 2-dimensional CV space.......................................19

Figure S16: Aromatic residues at the finger•little-finger interface in Y-family Pols.....................20

Figure S17: Deep-LDA framework 21

Figure S18: Selection of descriptors for the machine learning CVs. 22

Table 1: Sequence and structural alignment. 23

References 24

Supplementary Figures

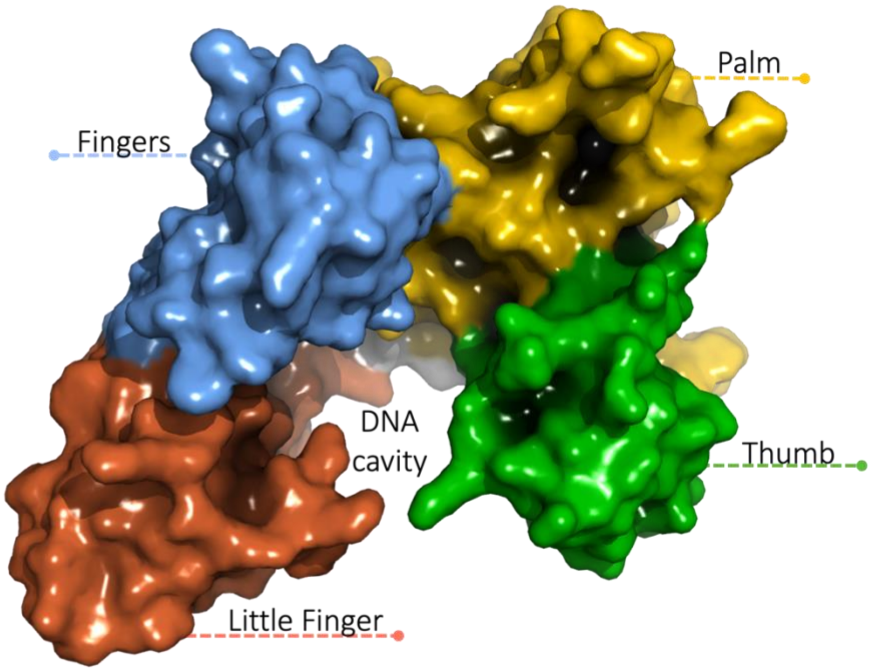

Figure S1: Structural representation of the Polη, highlighting its subdomain organization. The Fingers (blue), Palm (yellow), Thumb (green), and Little Finger (red) subdomains surround the DNA cavity, where the DNA substrate is accommodated. These subdomains play crucial roles in DNA binding, catalysis, and translocation during the polymerase function.

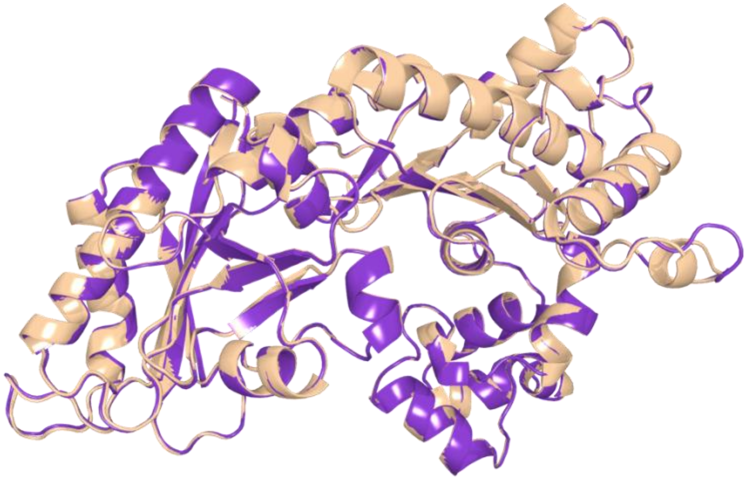

Figure S2: Structural superimposition of the catalytic core in pre-translocation (PDB ID 4ECX, wheat) and post-translocation (PDB ID 4ED8, purple) states X-ray structures.

The comparison revealed no major conformational changes between the two crystal structures (0.15 Å).[1]

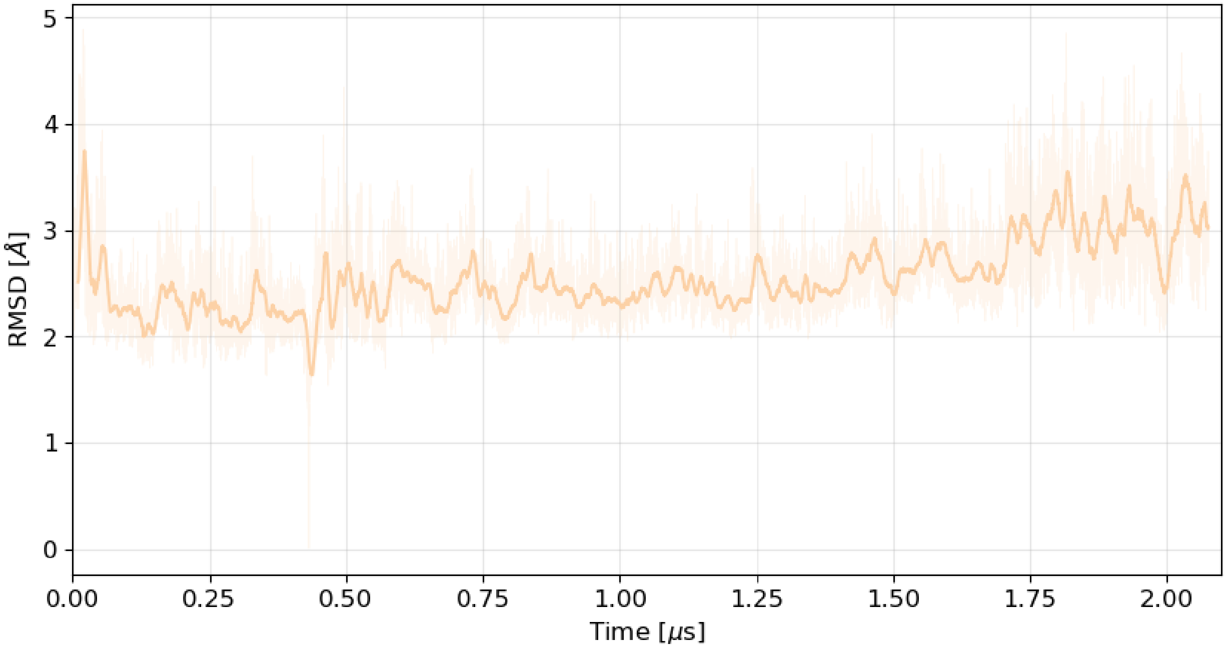

Figure S3: Time evolution of the Root-Mean-Square Deviation (RMSD) of the heavy atoms in the pre-translocation state over a 2 µs simulation.

The RMSD was calculated relative to the reference structure obtained from the first cluster of a dpeak clustering analysis.[2,3] All frames were aligned to the backbone atoms of the palm subdomain, excluding the loop regions. The solid pink line represents the rolling average of the RMSD using a window size of 100 frames, while the shaded area displays the complete dataset, highlighting the extent of structural fluctuations throughout the simulation. The gradual increase of the RMSD value around $\sim$1.70 μs is the starting point of a structural rearrangement of the system or a transition toward a different conformational state.

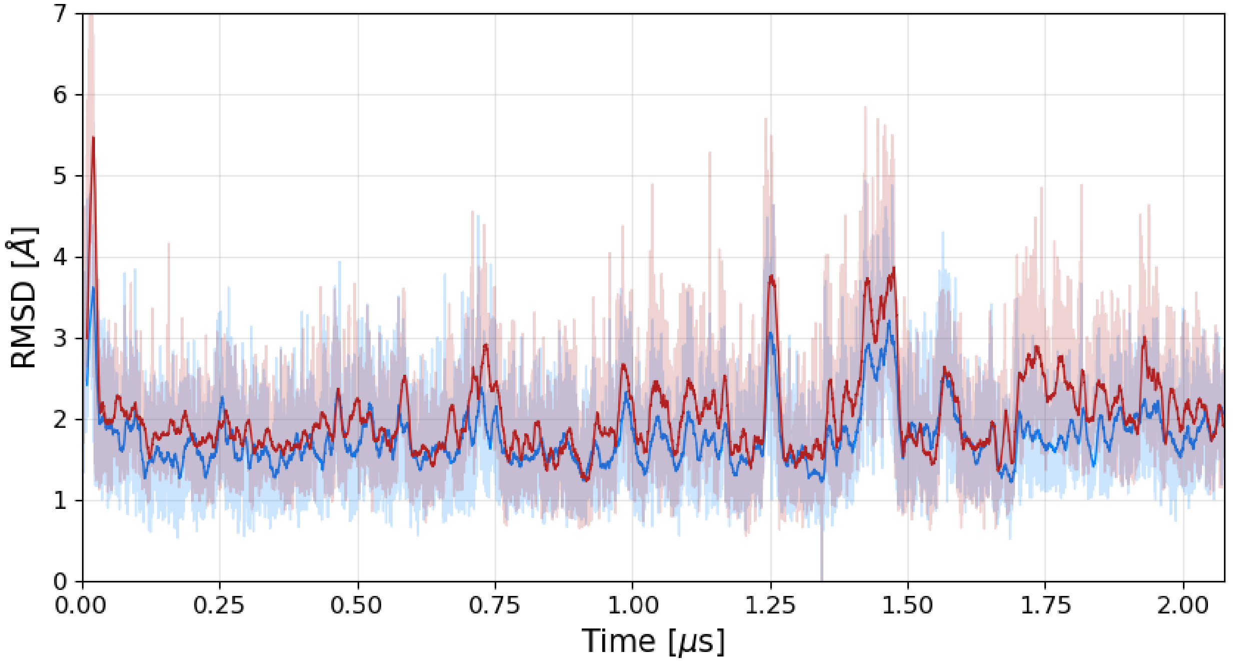

Figure S4: Root-Mean-Square-Deviation (RMSD) of the template strand (blue) and the primer strand (red) in the pre-translocation state.

The RMSD was calculated relative to the reference structure obtained from the first cluster of a *dpeak* clustering analysis.[2,3] All frames were aligned to the backbone atoms of the palm subdomain, excluding the loop regions. The RMSD showed is referring to the phosphate atoms of the double strand DNA. The solid pink line represents the rolling average of the RMSD using a window size of 50 frames, while the shaded area displays the complete dataset, highlighting the extent of structural fluctuations throughout the simulation.

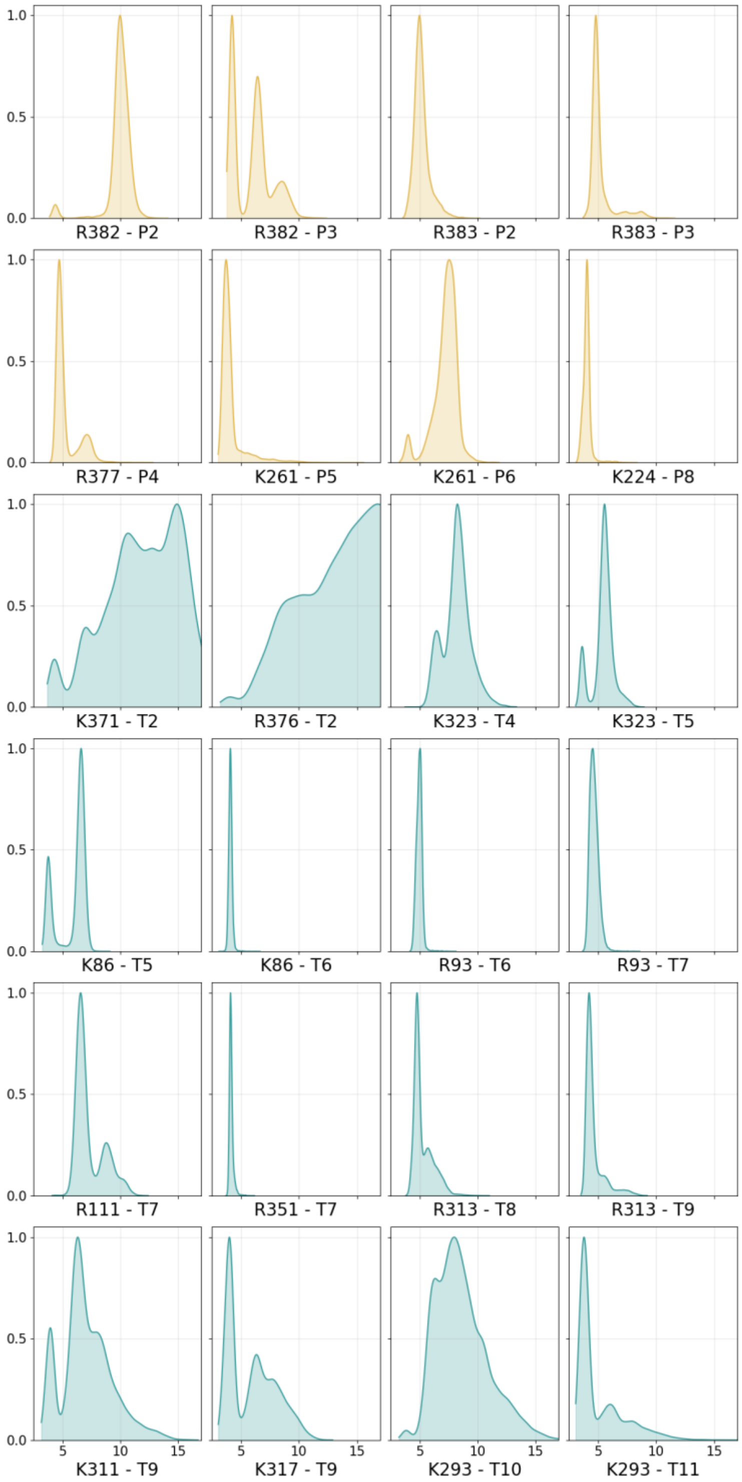

Figure S5: Probability distributions of key residue–DNA distances in the pre-translocation state.

Distances for primer-side interactions (gold) and template-side interactions (teal) are shown. All the distances are calculated with respect to the phosphate atom of the DNA substrate. The

distances involving a lysine are calculated using the nitrogen atom of the side chain. The distances involving an arginine are calculated using the carbon atom of the guanidinium group.

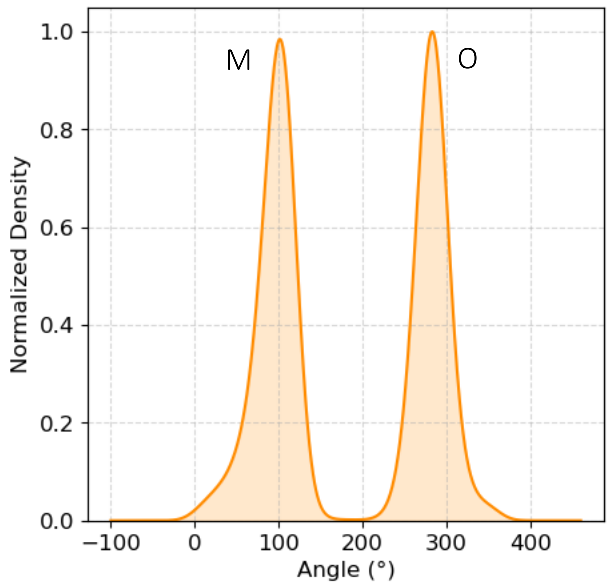

Figure S6: Pseudoihedral angle of Glu347 in the pre-translocation state classical MD simulation.

In the pre-translocation state MD simulation, Glu347 adopts two different conformations: inner (I), and outer (O). The inner conformation ($\sim$ 100°), Glu347 is bonded to Lys86, and Lys86 is bonded to T5. In the outer conformation ($\chi 1$ $\sim$ 300°), Glu347 completely flips outside, breaking the interaction with Lys86. At this point, Lys86 interacts with T6.

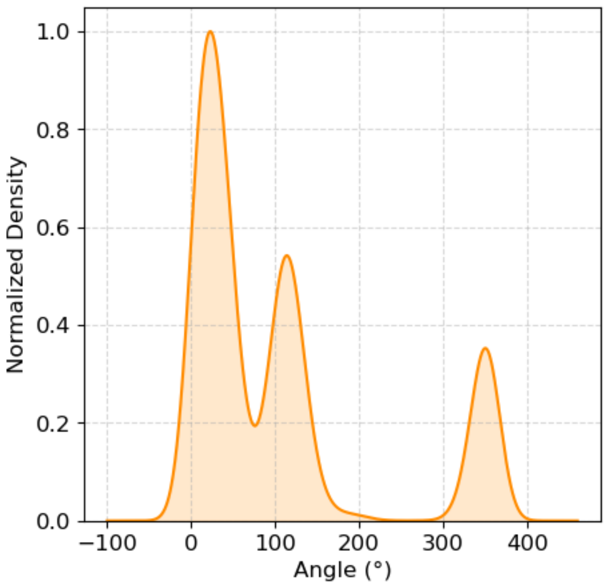

Figure S7: Probability density distribution of the pseudodihedral angle of Arg111 in the pre-translocation state.

The plot shows three distinct peaks around ~ 110° (DNA-bonded conformation) and the other two, ~ 20° and ~ 350° (both corresponding to the protein-bonded conformation), indicating frequent flipping of the Arg111 side chain. This conformational flexibility enables transient interactions with phosphate T7 via a bridging water molecule (6.6 ± 0.4 Å) or with Ser96 (distance measured with OH = 3.6 ± 0.2 Å), and occasionally with phosphate T8 through a water-mediated interaction (6.4 ± 0.5 Å).

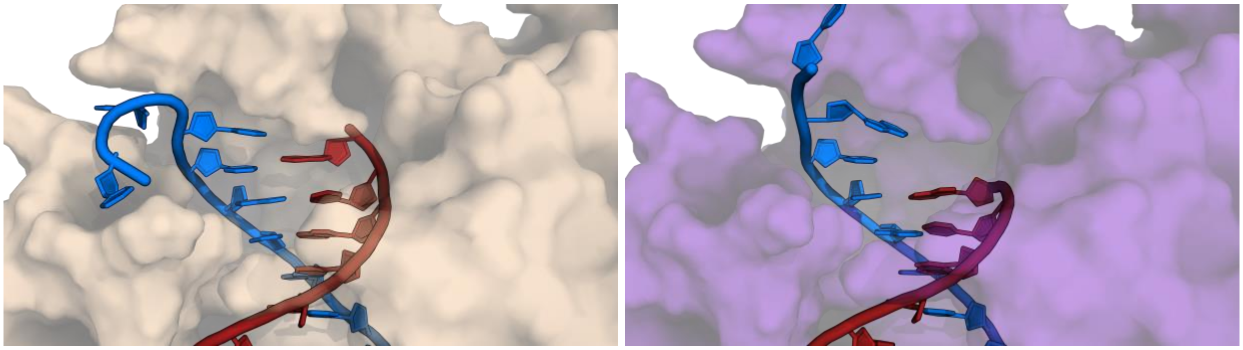

Figure S8: Comparison between the pre- and post-translocation states active site.

The protein is shown in surface and the DNA substrate in cartoon. The template strand is colored in blue, and the primer strand is in red. On the left, the pink protein referred to the pre-translocation state X-ray structure (PDB ID: 4ECX); on the right, the purple protein referred to the post-translocation state X-ray structure (PDB ID: 4ED8).[1] The pictures underline the fact that the active site in the post-translocation state is empty and can accommodate a new nucleotide.

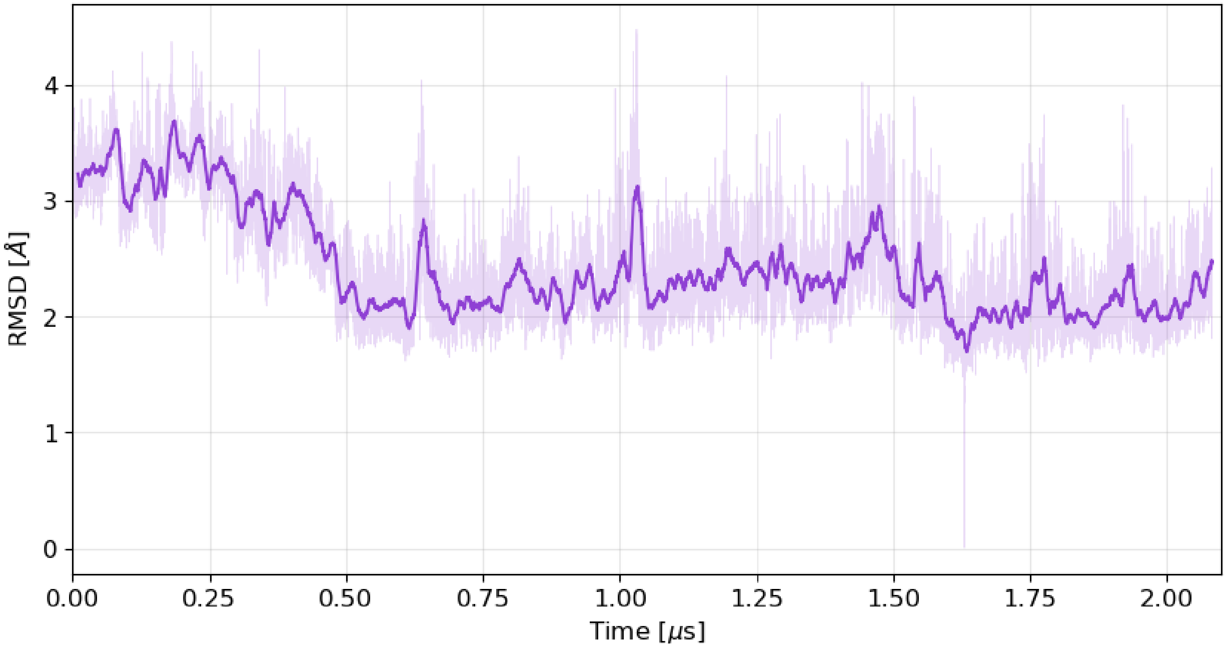

Figure S9: Time evolution of the Root-Mean-Square Deviation (RMSD) of the heavy atoms in the post-translocation state over a 2 µs simulation.

The RMSD was calculated relative to the reference structure obtained from the first cluster of a *dpeak* clustering analysis.[2,3] All the frames were aligned to the backbone atoms of the palm subdomain, excluding the loop regions. The solid purple line represents the rolling average RMSD using a window of 100 frames, while the shaded area displays the complete dataset, highlighting the extent of structural fluctuations throughout the simulation. The stabilization around ~2.2 Å suggests the system reaches a relatively stable conformation, with transient spikes indicating brief structural fluctuations or conformational changes.

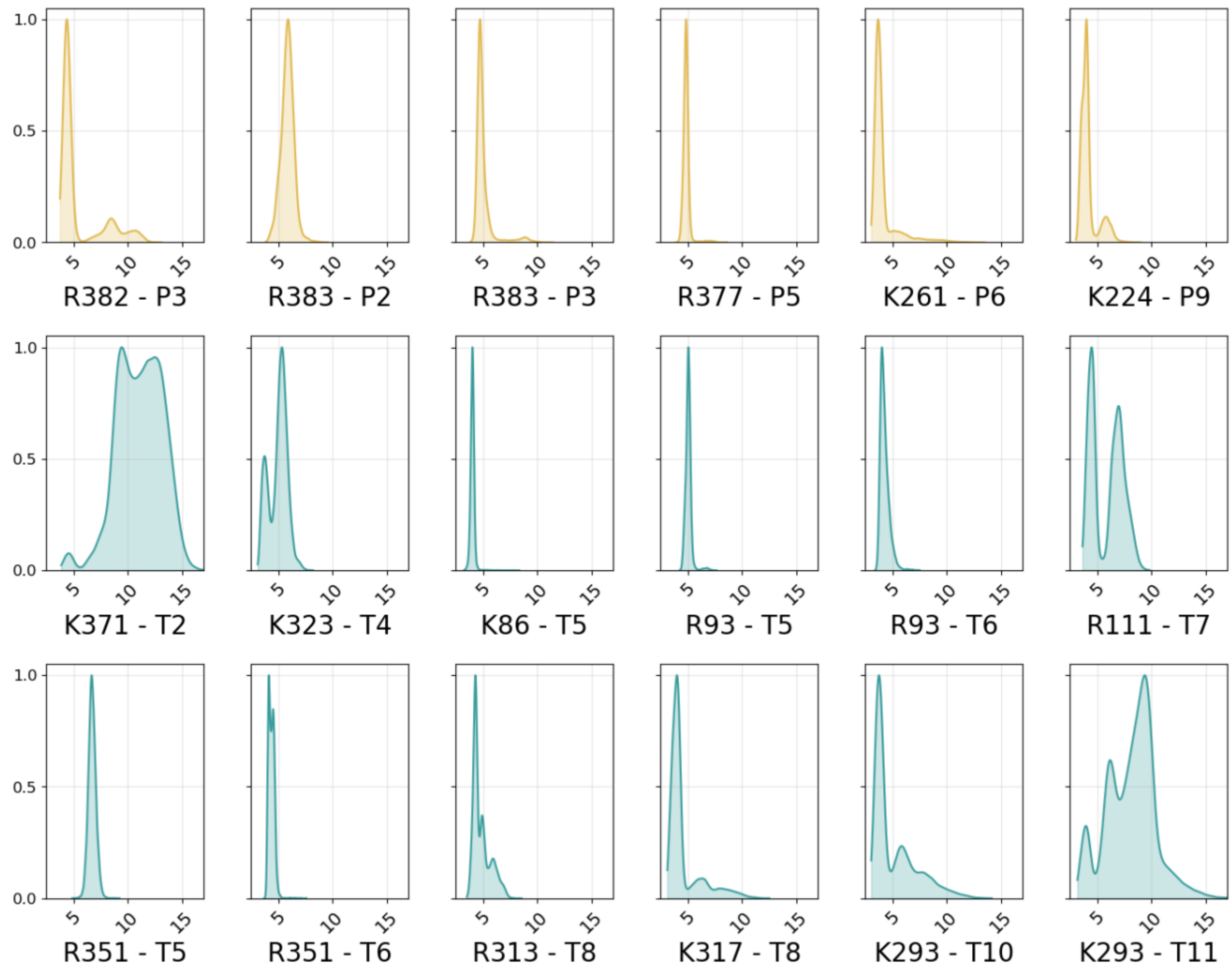

Figure S10: Probability distributions of key residue–DNA distances in the post-translocation state.

Distances for primer-side interactions (gold) and template-side interactions (teal) are shown. All the distances are calculated with respect to the phosphate atom of the DNA substrate. The distances involving a lysine are calculated using the nitrogen atom of the side chain. The distances involving an arginine are calculated using the carbon atom of the guanidinium group.

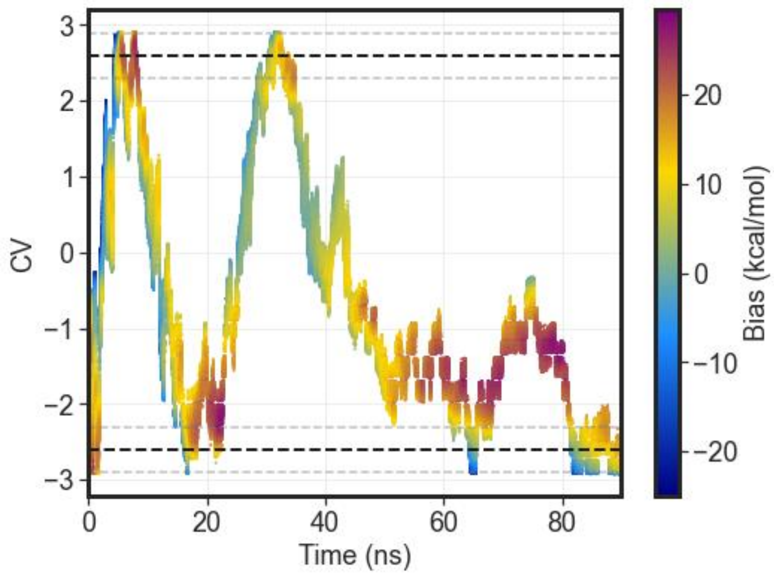

Figure S11: Evolution of the On-the-fly Probability Enhanced Sampling (OPES) 1D collective variable (CV) over time.[4–6]

Here, the pre-translocation state corresponds to the CV value-2.6, while the post-translocation state corresponds to the CV value +2.6. In this example, the system was able to translocate four times in ∼ 90 ns. The value set for BARRIER was 25 kcal/mol, and the color bar on the right represents the bias deposited during the simulation.

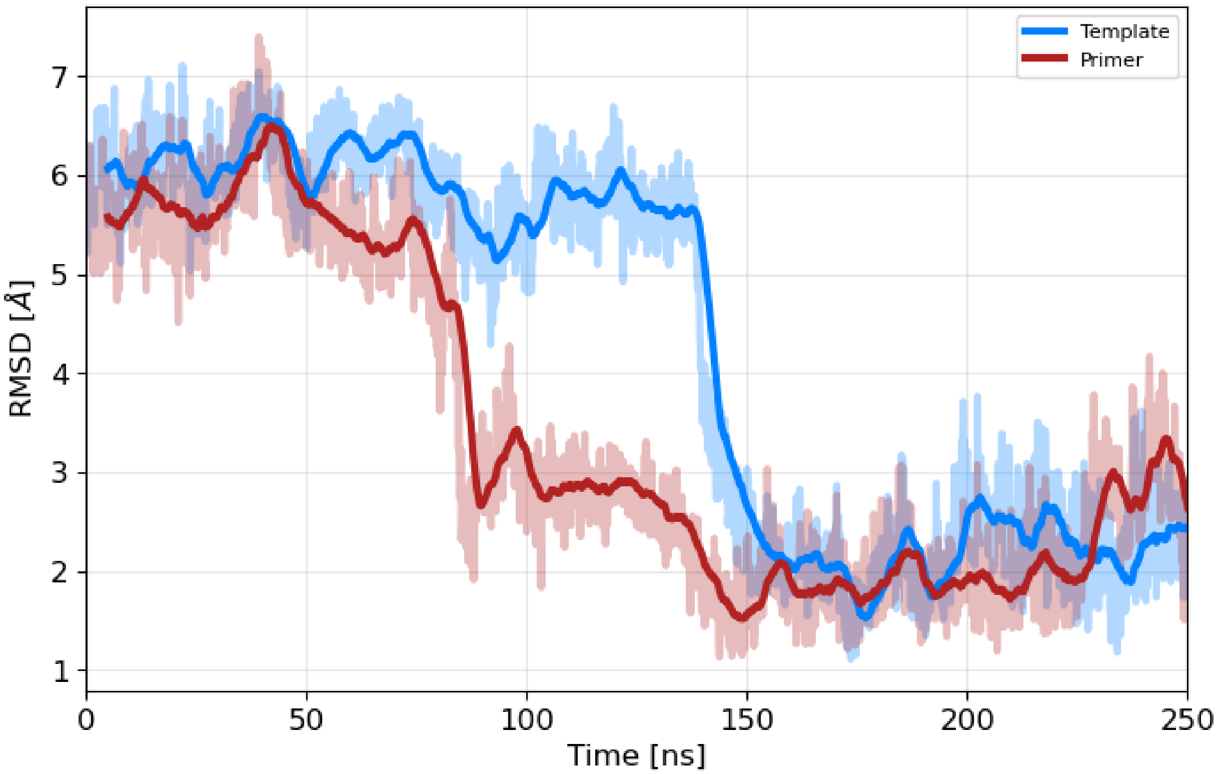

Figure S12: Root-Mean-Square-Deviation (RMSD) of the template strand (blue) and the primer strand (red) over time during the translocation.

In this simulation, the system was able to translocate once from the pre-translocation to the post-translocation state. The RMSD is calculated considering the dsDNA phosphate atoms with respect to the post-translocation state to appreciate the decrease in value, indicating the arrival at the correct final state. Here, the primer strand translocates ∼ 50 ns before the template strand, indicating an asynchronous mechanism for DNA translocation.

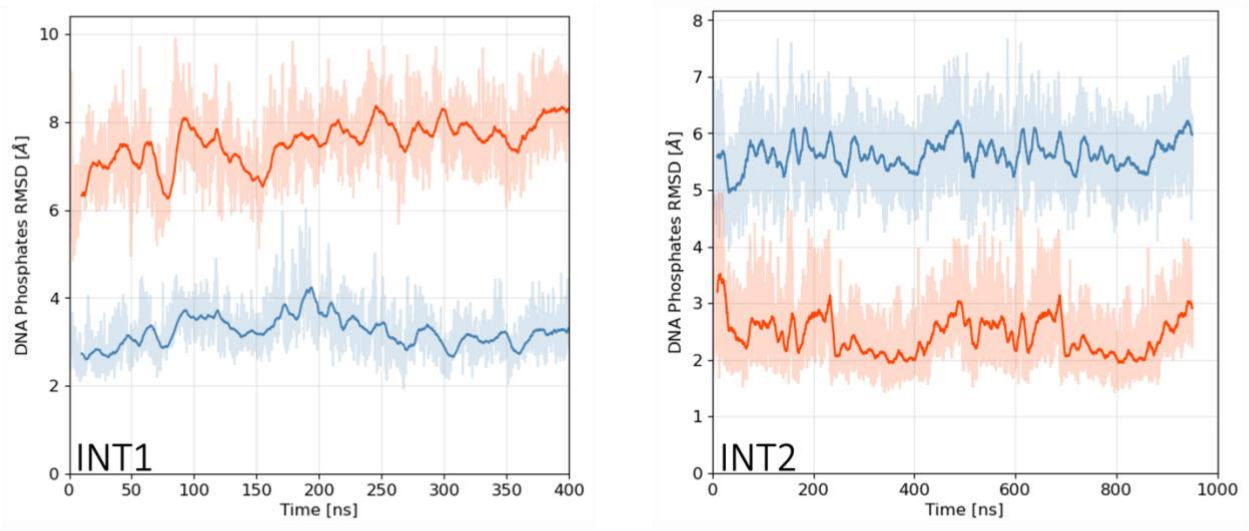

Figure S13: Classical MD simulations of the two intermediate states.

We calculated the RMSD of the template (blue) and the primer (red) strand of the two intermediate states INT1 and INT2, to check if they were stable. In both cases, the RMSD is calculated considering the dsDNA phosphate atoms with respect to the pre-translocation state, to appreciate the difference in value of the two strands.

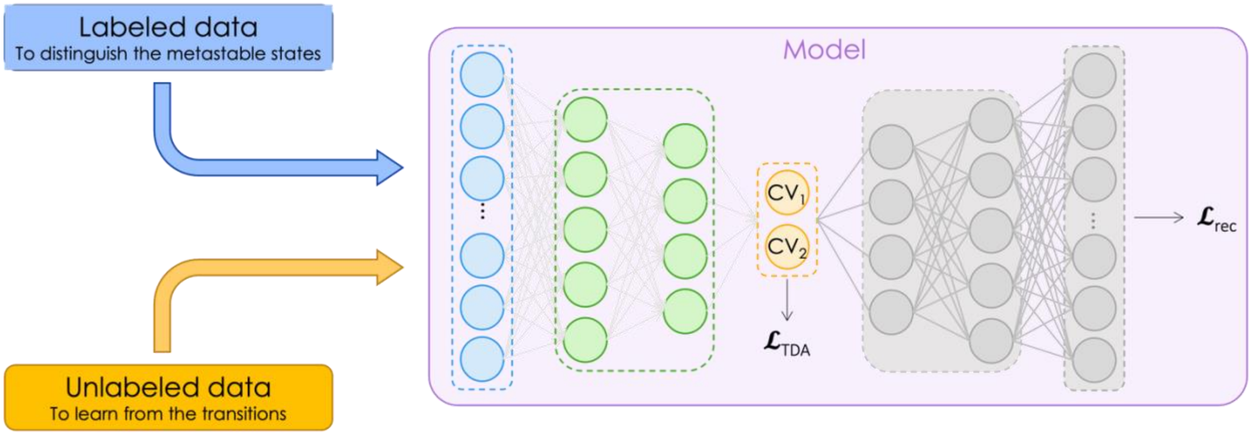

Figure S14: Multi-Task framework

Schematic representation of the multi-task framework used to design the 2-dimensional CV.[7] Here, we used as input the data coming from both the classical MD simulations (labeled data) and the previous transitions collected using the Deep-LDA CV (unlabeled data).[8] In this way, the model can learn how to distinguish the metastable states from the classical MD simulations, but also how the system has followed the path in the previous transitions.

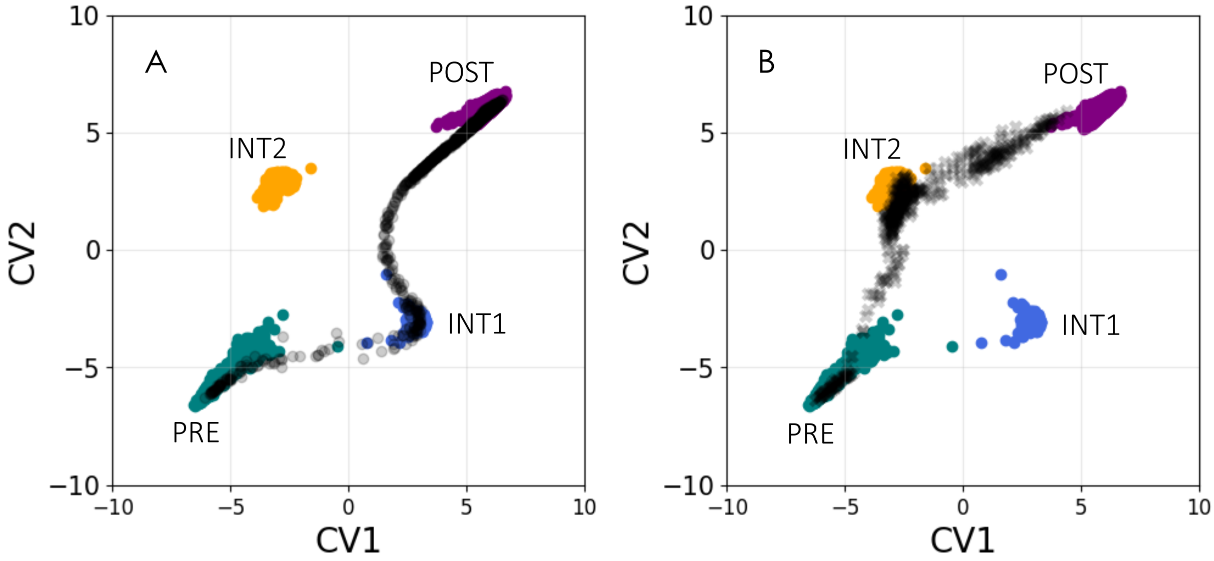

Figure S15: Two transition pathways in the 2-dimensional CV space.

(A) An example of path 1 followed by the system during one transition. Here, the system can reach POST from PRE by passing through INT1. (B) An example of path 2 followed by the system during one transition. Here, the system can reach POST from PRE by passing through INT2.

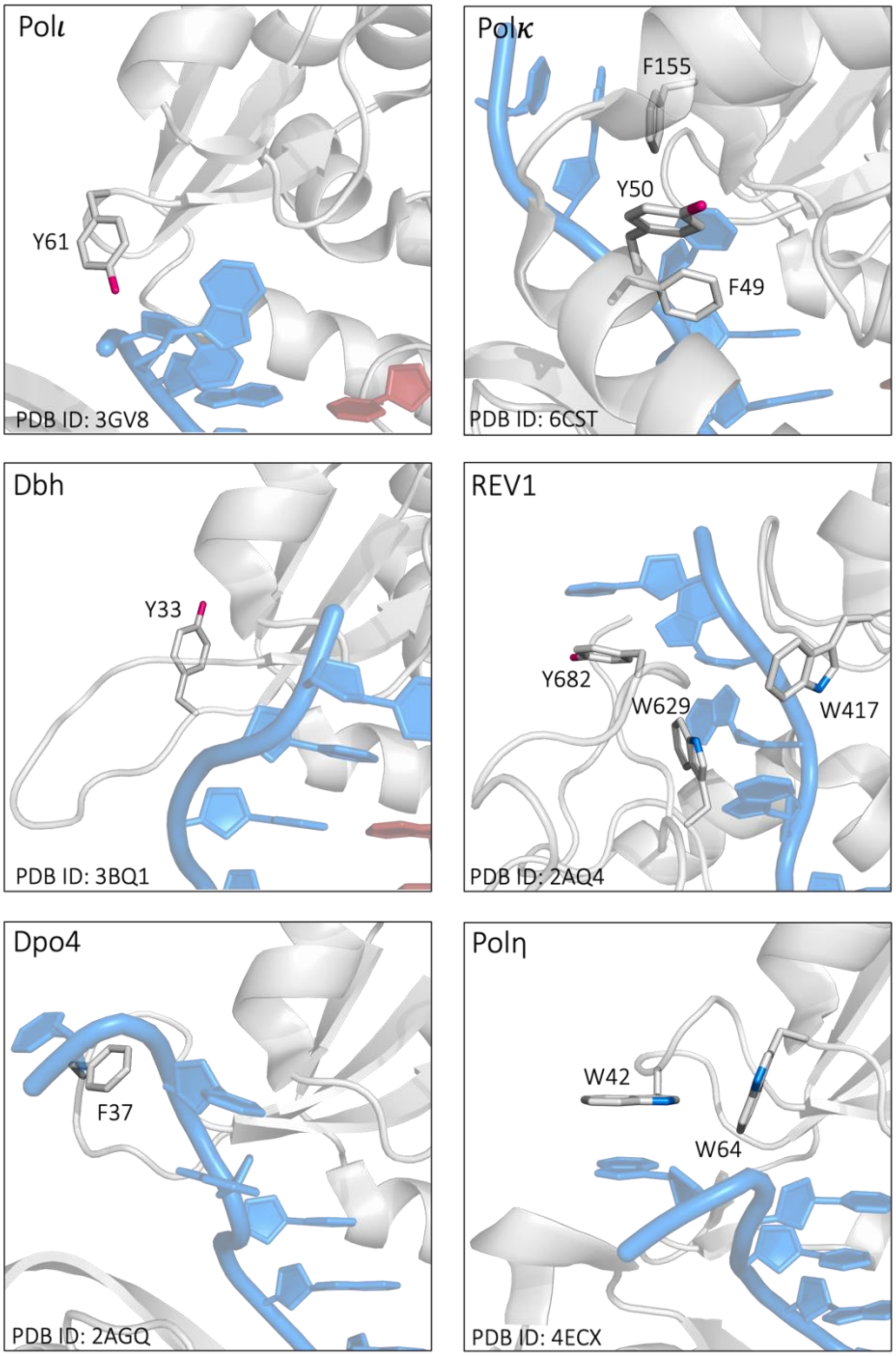

Figure S16: Aro**matic residues at the finger•little-finger interface in Y-family Pols.**

Zoom of the interface between finger and little-finger subdomain of all the six Pols belonging to the Y-family (Polι, 3GV8; Polκ, 6CST; Dbh, 3BQ1; REV1, 2AQ4; Dpo4, 2AGQ and Polη, 4ECX). In cartoon the protein (white) and the DNA substrate (the template strand in blue, the primer strand in red). In sticks, we underlined the aromatic residues present at this interface. In some cases, they can form stacking interactions with the template strand.

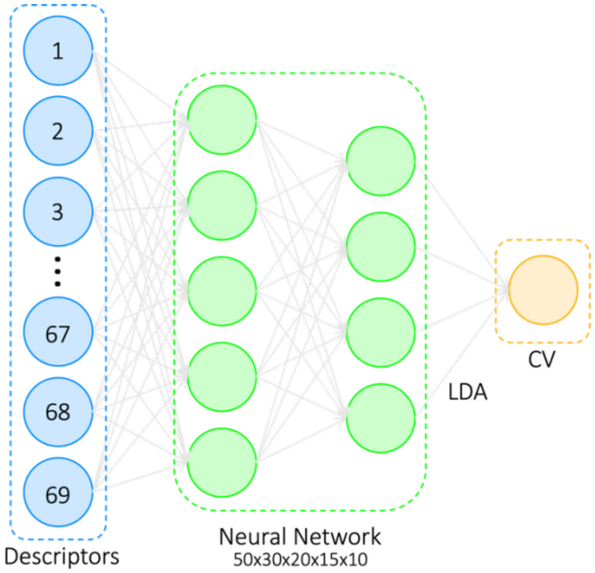

Figure S17: Deep-LDA framework

Schematic representation of the Deep-LDA framework used to design the 1-dimensional CV.[8] The building of the Deep-LDA collective variable (orange) involves using a set of physical descriptors (blue) as inputs to the neural network (green). The neural network applies a nonlinear transformation through multiple hidden layers. In the final layer, a linear discriminant analysis (LDA) is performed to identify the direction that maximizes the separation between classes, resulting in the Deep-LDA CV (orange).

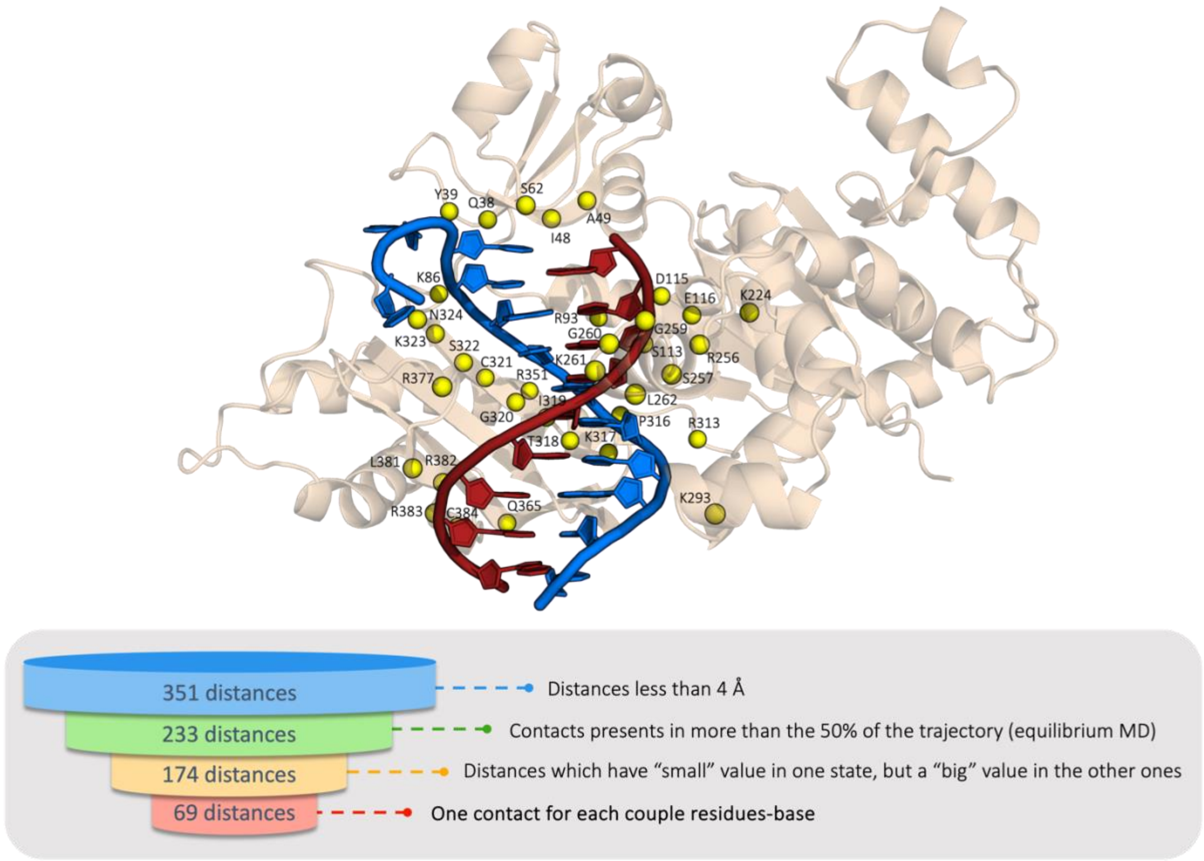

Figure S18: Selection of descriptors for the machine learning CVs.

The figure above illustrates the interaction network between the protein (light beige cartoon) and the nucleic acid (template in blue and primer in red), with yellow spheres representing the residue-base contacts selected as descriptors. The inset diagram summarizes the descriptor selection process: 351 distances correspond to all protein-DNA distances less than 4 Å. From these, 233 distances are retained as they are present in more than 50% of the equilibrium MD trajectory. A subset of 174 distances is identified, characterized by having “small” values in one state and “large” values in the other. Finally, 69 distances are selected, representing one contact for each residue-base pair.

Supplementary Tables

PDB ID	Sequence	Structure
2AGQ	2.3 Å	2.1 Å
2AQ4	3.0 Å	10 Å
3GV8	1.0 Å	7.1 Å
6CST	3.7 Å	8.6 Å
3BQ1	4.6 Å	4.9 Å

Table 1: Sequence and structural alignment.

Comparison of sequence and structural alignment for various PDB entries. The table reports the sequence alignment RMSD (in Å) in the middle column and the structural alignment RMSD (in Å) in the right column for each PDB ID.

References

(1) Nakamura, T.; Zhao, Y.; Yamagata, Y.; Hua, Y. J.; Yang, W. Watching DNA Polymerase η Make a Phosphodiester Bond. *Nature* **2012**, *487*, 196–201. https://doi.org/10.1038/nature11181.

(2) Rodriguez, A.; Laio, A. Clustering by Fast Search and Find of Density Peaks. *Science* **2014**, *344* (6191), 1492–1496. https://doi.org/10.1126/science.1242072.

(3) Case, D. A.; Aktulga, H. M.; Belfon, K.; Ben-Shalom, I. Y.; Brozell, S. R.; Cerutti, D. S.; Cheatham, T. E.; III; Cisneros, G. A.; Cruzeiro, V. W. D.; Darden, T. A.; Duke, R. E.; Giambasu, G.; Gilson, M. K.; Gohlke, H.; Goetz, A. W.; Harris, R.; Izadi, S.; Izmailov, S. A.; Jin, C.; Kasavajhala, K.; Kaymak, M. C.; King, E.; Kovalenko, A.; Kurtzman, T.; Lee, T. S.; LeGrand, S.; Li, P.; Lin, C.; Liu, J.; Luchko, T.; Luo, R.; Machado, M.; Man, V.; Manathunga, M.; Merz, K. M.; Miao, Y.; Mikhailovskii, O.; Monard, G.; Nguyen, H.; O'Hearn, K. A.; Onufriev, A.; Pan, F.; Pantano, S.; Qi, R.; Rahnamoun, A.; Roe, D. R.; Roitberg, A.; Sagui, C.; Schott-Verdugo, S.; Shen, J.; Simmerling, C. L.; Skrynnikov, N. R.; Smith, J.; Swails, J.; Walker, R. C.; Wang, J.; Wei, H.; Wolf, R. M.; Wu, X.; Xue, Y.; York, D. M.; Zhao, S.; Kollman, and P. A. Amber 2021. **2021**.

(4) Invernizzi, M.; Parrinello, M. Rethinking Metadynamics: From Bias Potentials to Probability Distributions. *Journal of Physical Chemistry Letters* **2020**, *11*, 2731–2736. https://doi.org/10.1021/acs.jpclett.0c00497.

(5) Invernizzi, M.; Parrinello, M. Exploration vs Convergence Speed in Adaptive-Bias Enhanced Sampling. *Journal of Chemical Theory and Computation* **2022**, *18*, 3988–3996. https://doi.org/10.1021/acs.jctc.2c00152.

(6) Trizio, E.; Rizzi, A.; Piaggi, P. M.; Invernizzi, M.; Bonati, L. Advanced Simulations with PLUMED: OPES and Machine Learning Collective Variables. *arXiv* **2024**. https://doi.org/10.48550/arxiv.2410.18019.

(7) Bonati, L.; Trizio, E.; Rizzi, A.; Parrinello, M. A Unified Framework for Machine Learning Collective Variables for Enhanced Sampling Simulations: Mlcolvar. *Journal of Chemical Physics* **2023**, *159*. https://doi.org/10.1063/5.0156343.

(8) Bonati, L.; Rizzi, V.; Parrinello, M. Data-Driven Collective Variables for Enhanced Sampling. *Journal of Physical Chemistry Letters* **2020**, *11*, 2998–3004. https://doi.org/10.1021/acs.jpclett.0c00535.
