# Supplementary material for: Coordinated Residue Motions at the Enzyme–Substrate Interface Promote DNA Translocation in Polymerases
Source: J Am Chem Soc. 2025 Jun 17;147(26):22972–85. doi: 10.1021/jacs.5c05888 (PMC12232177; doi:10.1021/jacs.5c05888)
Supplement: Supplementary file 2 [file ja5c05888_si_002.pdf]

Movie 1: Conformational Transitions of Human DNA Polymerase η During DNA Translocation. This movie illustrates the structural dynamics of human DNA polymerase η (Polη, beige cartoon) as it progresses through pathway 1 (PRE, INT1, and POST states), highlighting molecular rearrangements that facilitate DNA translocation. Here, the trajectory starts with the system in the pre-translocation state (PRE). At ~13 s, the system begins the transitions from the PRE state to the intermediate state INT1, in which the primer strand (red cartoon) translocates by one base pair. Subsequentially, the primer translocation is followed by the template strand (blue cartoon) translocation at ~37 s, and the system eventually reaches the post-translocation state (POST).

Movie 2: Asynchronous Translocation Pathway — Zoom on Arg382 and Arg383 Conformational Rearrangements. This movie provides a close-up view of the asynchronous DNA translocation mechanism, focusing on the rearrangements of Arg382 (left) and Arg383 (right). Our simulations reveal significant side chain reorganization of positively charged residues at the Polη•DNA interface that promote DNA translocation along two possible pathways, characterized by two different intermediate states (either INT1 or INT2). In this movie, we depicted 2 out of 17 positively charged residues at the Polη•DNA interface for the sake of clarity. Only the trajectory passing through INT1 is exhibited. The protein is displayed in beige cartoon, while the DNA template and primer strands, are in blue and red cartoons, respectively. Arg382 and Arg383 side chains are shown as sticks.
