# Supplementary material for: Coordinated Residue Motions at the Enzyme–Substrate Interface Promote DNA Translocation in Polymerases
Source: J Am Chem Soc. 2025 Jun 17;147(26):22972–85. doi: 10.1021/jacs.5c05888 (PMC12232177; doi:10.1021/jacs.5c05888)
Supplement: Supplementary file 5 [file ja5c05888_si_005.zip › Additional Supporting Information.docx]

Additional Supporting Information

**Coordinated residue motions at the enzyme-substrate interface promote DNA translocation in polymerases.**

Alessia Visigalli, Enrico Trizio2 Luigi Bonati, Pietro Vidossich, Michele Parrinello, Marco De Vivo

**Structure files (PDB):**

The following structures correspond to the **centroids of the most populated clusters** obtained via k-medoids clustering of the MD trajectories:

- Pre_translocation.pdb – Centroid structure of the pre-translocation state
- Post_translocation.pdb – Centroid structure of the post-translocation state
- Intermediate1.pdb – Centroid structure of the first intermediate (INT1)
- Intermediate2.pdb – Centroid structure of the second intermediate (INT2)

**Input files:**

These files were used for the enhanced sampling simulations involving the 2D collective variable (multi-task CV):

- plumed.dat – Main PLUMED input file for enhanced sampling simulations
- plumed_descriptors.dat – DNA•protein distances used as descriptors for enhanced sampling simulations
- plumed_rst.dat – Restraint parameters to maintain hydrogen bonds between the DNA bases during the simulations

**Output files:**

These are the output files generated from the enhanced sampling simulations with the 2D collective variable (CV) (see the input files above):

- COLVAR – OPES parameters used for the enhanced sampling simulations.
- DIST – DNA•protein distances used as descriptors for the machine-learning CV calculated over the biased trajectory
- FES.dat – Free energy surface (FES) derived from enhanced sampling simulations
